# Asháninka medicinal plants: a case study from the native community of Bajo Quimiriki, Junín, Peru

**DOI:** 10.1186/1746-4269-6-21

**Published:** 2010-08-13

**Authors:** Gaia Luziatelli, Marten Sørensen, Ida Theilade, Per Mølgaard

**Affiliations:** 1 Department of Agriculture and Ecology, University of Copenhagen, Rolighedsvej 21, DK-1958 Frederiksberg C, Denmark; 2 Forest and Landscape, University of Copenhagen, Rolighedsvej 23, DK-1958 Frederiksberg C, Denmark; 3 Department of Medicinal Chemistry, University of Copenhagen, Universitetsparken 2, DK-2100 Copenhagen, Denmark

## Abstract

**Background:**

The Asháninka Native Community Bajo Quimiriki, District Pichanaki, Junín, Peru, is located only 4 km from a larger urban area and is dissected by a major road. Therefore the loss of traditional knowledge is a main concern of the local headman and inhabitants. The present study assesses the state of traditional medicinal plant knowledge in the community and compares the local pharmacopoeia with the one from a related ethnic group.

**Methods:**

Fieldwork was conducted between July and September 2007. Data were collected through semi-structured interviews, collection of medicinal plants in the homegardens, forest walks, a walk along the river banks, participant observation, informal conversation, cross check through voucher specimens and a focus group interview with children.

**Results:**

Four-hundred and two medicinal plants, mainly herbs, were indicated by the informants. The most important families in terms of taxa were Asteraceae, Araceae, Rubiaceae, Euphorbiaceae, Solanaceae and Piperaceae. Eighty-four percent of the medicinal plants were wild and 63% were collected from the forest. Exotics accounted to only 2% of the medicinal plants. Problems related to the dermal system, digestive system, and cultural belief system represented 57% of all the medicinal applications. Some traditional healers received non-indigenous customers, using their knowledge as a source of income. Age and gender were significantly correlated to medicinal plant knowledge. Children knew the medicinal plants almost exclusively by their Spanish names. Sixteen percent of the medicinal plants found in this community were also reported among the Yanesha of the Pasco Region.

**Conclusions:**

Despite the vicinity to a city, knowledge on medicinal plants and cultural beliefs are still abundant in this Asháninka Native Community and the medicinal plants are still available in the surroundings. Nevertheless, the use of Spanish names for the medicinal plants and the shift of healing practices towards a source of income with mainly non-indigenous customers, are signs of acculturation. Future studies on quantification of the use of medicinal plants, dynamics of transmission of ethno-medicinal knowledge to the young generations and comparison with available pharmacological data on the most promising medicinal plants are suggested.

## Background

Peru is one of the twelve most biodiversity rich, or 'mega-diverse', countries of the world. Its combination of latitude and topography creates numerous ecosystems which are home to an extraordinarily rich flora and fauna. At least 25,000 species of plants, of which 5,354 are endemic, are hosted in its numerous ecosystems [1]. The country is also highly diverse in cultures represented by its many ethnic groups: according to the last census from 2007 the indigenous population of the Peruvian Amazon consists of approximately 333,000 individuals, belonging to 59 ethnic groups and 15 linguistic families. The largest ethnic group is the Asháninka with a population of almost 90,000 which represents 26% of the indigenous population recorded in the Peruvian Amazon [2].

Medicinal plants constitute an important resource to indigenous people, who often lack access to conventional health care systems either due to isolation or to economy. This is a common condition in developing countries: e.g. in some African and Asian countries, 80% of the population depend on traditional medicine for primary health care [3], while according to WHO Regional Office for the Americas 40% of the Colombian population and 71% of the Chilean population have used Traditional Medicine.

The Asháninka language belongs to the Arawak linguistic group and has affinities with the Piro, the Matsigenka and Yanesha languages in the Peruvian Amazon [4,5]. The Asháninka live in the foothills of the Andean region in the central part of Peru also known as 'Selva Alta' or 'Ceja de Selva', in the valleys of the Apurímac, Ene, Tambo, Satipo, Perené, Pichis and Pachitea rivers [4,5]. Literature on the Asháninka ethnic group has been produced mainly in the form of ethnographic studies [4,6], while ethnobotanical studies are scarce. Existing ethnobotanical studies have so far been concentrated either in the Peruvian Amazons or 'Selva Baja' in the Departments of Loreto, Ucayali and Madre de Dios [7-24] or in the Peruvian Andes [25-27]. One of the few ethnobotanical studies specific to the Asháninka of the Peruvian Selva Central was published 20 years ago reported 96 species of medicinal plants indicated by 4 informants in 5 indigenous communities [5]. Keplinger *et al*. [28] present a brief description of the Asháninka medical system before focusing on the ethnomedicinal uses and pharmacological results of *Uncaria tomentosa *(Willd.) DC.,'uña de gato' (cat's claw) which is widely known and used by the Asháninka.

A more recent study conducted in four Asháninka communities, of which three in the Ucayali Department of Peru and one in the Brazilian state of Acre, reported interesting results on the structure, transmission and transformation of environmental knowledge in these communities [22,29]. Lenaerts [30] describes the ethno-medicine and in particular the relation between body and environment and inter-ethnic borrowing by the Ashéninka of the Ucayali and Ashéninka del Gran Pajonal, who are related to the Asháninka. However, this study does not include any details regarding plant identity with the stated purpose of protecting the indigenous intellectual property rights. Bletter [23] has proposed a new quantitative theoretical framework for discovering plant-derived medicines based on the hypothesis that "closely related plants used to treat closely related diseases in distantly related cultures have a higher probability of being effective". He compared the families and genera of the medicinal plants used by the Asháninka of Peru and the Malinké of Mali against eight diseases and found a significant similarity between the two medicinal floras, thought only if the diseases were grouped into the categories of parasitic and autoimmune diseases. Sosnowska and Balslev [31] recently published a comprehensive review of the American palms used in local traditional medicine, based on literature from the last 25 years, which included also data on the Asháninka of Peru.

The Native Community of Bajo Quimiriki is located at the banks of the Perené River in the Peruvian Department of Junín. Here the majority of the population relies exclusively on medicinal plants for self-medication. This is due to the free availability of the resource, cultural traditions and cost of hospital treatments in the nearby town of Pichanaki. The proximity to Pichanaki does constitute a threat for the future survival of the indigenous knowledge and practicesas the younger generations are more and more assimilated by the dominant society. The children of the community spend most of the day at school, where they are taught in Spanish. This decreases their chances to learn about the uses of the medicinal plants from the older people. Several [18,32,33] studies demonstrate that medicinal plants lore is particularly vulnerable to acculturation and the ethno-cultural erosion due to globalization is discussed in numerous published ethnobotanical studies [25,27].

The aim of the study was to document the medicinal tradition, thus contributing to an increased understanding of the distribution of knowledge among the community inhabitants, and to record the practices related to medicinal plant preparation and administration. Medicinal plant habitats and the frequency and use of cultivated and exotic plants were also investigated.

## Methods

### Study area

**Figure 1:**
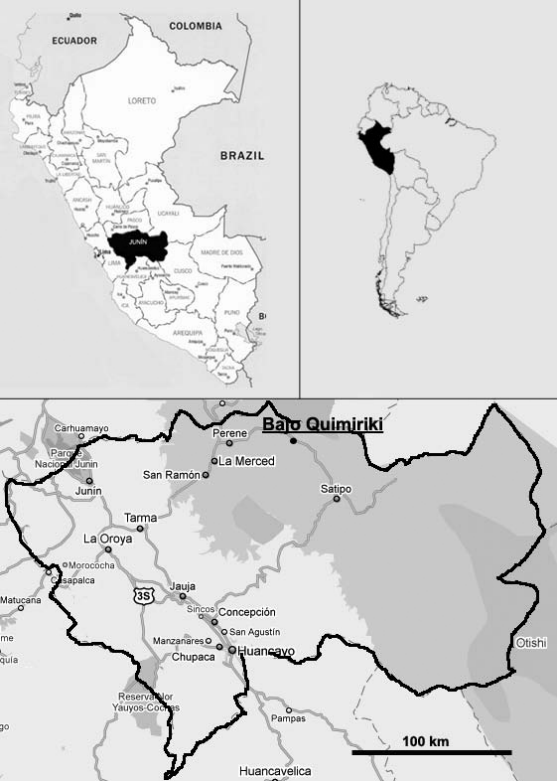
**Map of the study area**. The map shows the location of the Native Community Bajo Quimiriki. The community is crossed by a major road, which connects the cities of La Merced and Satipo.

Bajo Quimiriki is located in the District of Pichanaki, Province Chanchamayo, Department Junín, at the oriental foothills of the Andes, with coordinates 10°56' S and 74°51' W (Figure 1). The distance to the neighbouring town Pichanaki is 4 km, along the paved road that follows the river Perené connecting Pichanaki to the city of Satipo. The community covers an area of 268 ha and the altitude varies from 400 m a.s.l. along the basin of the Perené river to 900 m in the forest covered hills. However, most households are located along the Marginal Road at approximately 515 m a.s.l. The climate corresponds to the tropical-humid forest according to Holdridge's classification [34]. The mean annual precipitation is 1500 mm, with main rainfall in January - March, whilst the driest months are June and July. The temperature during the year varies between 22°and 26°C [35].

The population of Bajo Quimiriki includes 37 indigenous households and 6 households of colonos (non-indigenous Peruvians who migrated in the area in search for land to cultivate and/or better opportunities). The hut-like houses are close to each other, usually with people belonging to the same family living in proximity. They have one or two levels built with wooden support poles, cane walls and palm frond thatching. Every household has a piece of land for cultivation called 'chacra', the extent of which varies from 2 to 6 ha. In the past the main cultivated crop was yuca (*Manihot esculenta *Crantz), a staple food consumed roasted or used to produce 'masato', a fermented alcoholic drink. Other popular plants were achiote (*Bixa orellana *L.) used to paint bodies and arrows, platano (*Musa paradisiaca *L.), pituca (*Xanthosoma sagittifolium *Schott.), various types of beans and citrics. Nowadays, the need for cash has led to an intensification of the work in the chacra, where cash crops such as cacao (*Theobroma cacao *L.) and coffee (*Coffea *sp.) have been introduced, while the cultivation of achiote and bananas (*Musa *sp.) has been intensified to be sold in the market. Due to the need for cash, the men occasionally work the agricultural land of colonos as day labourers, while the women spend more time tending the chacra than before. Hence, they give less priority to traditional crafts like handweaving of the traditional tunic called 'cushma'.

### Methods of collection of ethnobotanical information

The fieldwork was carried out during the period July-September 2007.

In the course of two community meetings the people of the community agreed on the subject of the study, on the methods to be used and on eventual economic compensations.

Data were collected through household interviews, collection of medicinal plants in the homegardens, forest walks, a walk along the river banks, participant observation, informal conversation, cross check through voucher specimens and a focus group interview with children.

The informants for the forest walk were selected either because they were indicated by the deputy-headman as most knowledgeable on medicinal plants (after the 'specialists' who could not or did not want to participate) or in a couple of cases because they declared to make use of medicinal plants and agreed to participate in the event.

We attempted as far as possible to have an equal number of female and male informants, having for example six men and six women both in the forest walks and in the cross check with voucher specimens, but this was not possible for the households interviews because men were difficult to find at home. The walk on the river bank was done with three women indicated by the deputy-headman.

We agreed on a compensation corresponding to a daily salary of an agricultural worker in the area for the participants to the forest walks, which took a whole day.

#### Household interviews

Interviews were carried out in sixteen of a total of thirty-seven indigenous households (43%). The informants were eleven women and five men. The households were chosen randomly. Semi-structured interviews were applied in order to present some important questions to all the households while remaining flexible as suggested by Cotton [36]. During the interviews the respondent's name, age, profession, place of birth, number of years spent in the community were recorded. Successively the respondents were asked who they believed were the most knowledgeable persons on medicinal plants in the household and in the community. The informants were also asked who they relied on in case of illness, e.g. self-medication, a healing specialist in the community, the doctor from nearby town, or others. Diseases that had occurred in the specific household were described, including information on causes, symptoms and their respective cure. Any herbal medicinal remedy stored in the house was recorded and its application described by the informant.

#### Collection of medicinal plants in the homegardens

A walk through the homegarden followed the household interview. The informants were asked to describe the medicinal application and preparation of any medicinal plant cultivated. Information was collected also on plants that were not actively cultivated but grew spontaneously in the proximity of the house and were indicated as medicinal.

#### Forest walks

In our study, differently from others [18,30,37-39] the ethnobotanical information on forest plants were collected in a no-random way, using forest walks rather than transects or quadrants with pre-selected species with DBH superior to 2.5, 5 or 10 cm. We chose to apply this method as we did not have parcels with already identified species in proximity of the community and we did not want to limit our study to trees and lianas with diameter at breast height (DBH) superior to 2.5, 5 or 10 cm as reported in numerous studies [18,30,37-39] because we knew that medicinal plants are often herbs. A path in the community forest reserve was established with the help of three men recommended by the deputy headman as knowledgeable on the forest and medicinal plants. Medicinal plants identified by the informants were marked. Successively the forest route was walked with twelve informants, six men and six women of different ages. Each informant was guided through the route on a separate day. Interviews were made at each marked plant. The informant would be asked whether he/she knew the plant, and if yes, the name would be recorded in both in Asháninka and Spanish. The medicinal use, plant parts used and preparation were also recorded. In addition to the marked plants, all informants recognised a number of additional medicinal plants on the track: these were also collected. The performances of men and women were compared by summing up for each gender the number of events in which a medicinal application and preparation for one plant was described on the track (if an informant reported more than one use for the same plant it counts as one record).

#### Walks on the river banks

Some informants indicated that a number of medicinal plants were collected along the banks of the Perené River, where some of the community members had their *chacra*. A one day trip with three women from the village was organized to collect the medicinal plants of that area as well as information on uses and preparation. The women who participated in this collection were aged 25, 35 and 36 and they all had children.

#### Cross check of information on 80 selected medicinal plants

The study focused on the most well-known plants in the community based on the assumption that best known plants are more likely to contain active compounds with ability to cure particular diseases. Hence, a subset of plants recognised by more than one informant was selected for further analysis. Twenty of the pre-marked plant species recognised as medicinal by at least six out of twelve informants were selected from the forest walk. An additional twenty not pre-marked species, identified in the forest by more than one informant, were selected. The same method was used for twenty plants collected in the homegardens. Twenty out of twenty-three plants collected at the river were chosen eliminating those that had already been collected elsewhere. These eighty plants were shown in the form of herbarium specimens to six women and six men that had not participated in the forest walks, in order to perform a cross check of the ethnobotanical information.

#### Focus group interview with children

A focus group interview was conducted with the children of the 5^th ^and 6^th ^grade of elementary school to discuss their interest in and knowledge of medicinal plants. The interview was undertaken during a school hour. The class was composed of 17 children, 9 girls and 8 boys. The children were asked to list the medicinal plants they knew by heart and their uses and preparation. The answers were given collectively. During this exercise no voucher specimens were collected and the plants were identified by the vernacular names provided by the children.

#### Preparation and identification of the voucher specimens

The plant material was pressed and dried in the field. One set of the collected plants was deposited in the herbarium USM in Peru and a second set of plants was deposited in the herbarium CP in Denmark. The plants were partly identified in the herbarium USM with the help of local specialists and students (see Acknowledgments) and partly in Denmark with the help of various floras [40-44], books dealing with the medicinal flora of Peru and South America [1,45-53] and digital herbaria [54,55]. These sources were also used to determine the taxa origin. All the collection numbers and author names are reported in Additional file 1 under the scientific name of the plant. The author's names follow the standard form by Brummitt and Powell [56].

## Results

### Medicinal plants - diversity, life form and habitat

A total number of 402 taxa were indicated as having medicinal properties by the informants when applying the different ethnobotanical methods. The plants have been identified to the following different taxonomic hierarchic level: species (208 plants), genus (93 plants), family (54 plants), not identified (47 plants) (See Additional file 1). The taxa identified to family level (355 plants), belong to 72 distinct families. The six most important families in terms of number of taxa were Asteraceae, Araceae, Rubiaceae, Euphorbiaceae, Solanaceae and Piperaceae (Table 1). The plants indicated as medicinal were mostly herbs, but the local inhabitants used also shrubs, trees, vines, epiphytes, lianas and ferns (Table 2).

**Table 1:** List of most important plant families in terms of species used as medicinal plants (families with at least three taxa)

Family	No. of taxa	No. of vouchers	%*
Asteraceae	31	44	12.4
Araceae	17	29	8.2
Rubiaceae	15	21	5.9
Euphorbiaceae	15	17	4.8
Solanaceae	13	17	4.8
Piperaceae	13	18	5.1
Verbenaceae	10	12	3.4
Fabaceae	9	10	2.8
Cyperaceae	9	9	2.5
Poaceae	8	11	3.1
Malvaceae	7	9	2.5
Commelinaceae	7	7	2.0
Urticaceae	6	8	2.3
Arecaceae	6	7	2.0
Acanthaceae	6	8	2.3
Bignoniaceae	5	5	1.4
Apocynaceae	5	7	2.0
Zingiberaceae	4	4	1.1
Melastomataceae	4	4	1.1
Gesneriaceae	4	4	1.1
Tiliaceae	3	3	0.8
Phytolaccaceae	3	4	1.1
Moraceae	3	3	0.8
Menispermaceae	3	6	1.7
Maranthaceae	3	3	0.8
Costaceae	3	3	0.8
Clusiaceae	3	3	0.8

* The percentage is calculated on the 355 herbarium samples that have been identified.			

**Table 2:** Life form

Life form	No. of taxa	%
Herb	209	65
Shrub	45	14
Tree	35	11
Liana	10	3
Small tree	7	2
Vine	7	2
Epiphyte	6	2
Arborescent fern	1	0.3

**Figure 2:**
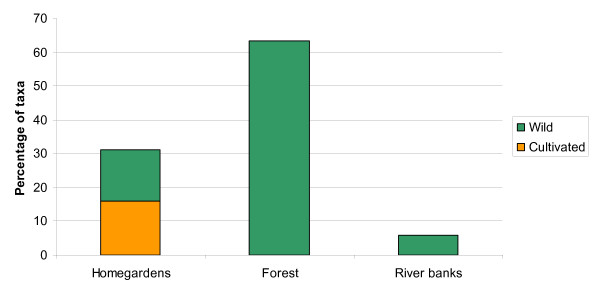
**Medicinal plants habitats**. Distribution to habitat of the 402 medicinal plants. A total of 63% of the species grow in the forest, 15% in the homegardens and 6% on the river banks. Eight taxa grow both in the homegardens and in the forest, two grow both in the homegardens and on the river banks and one grows in all the three habitats. Eighty-one percent of the species is wild and 16% cultivated.

The majority of the medicinal plants were found in the forest (63%), followed by the homegardens (31%) and the river banks (6%). Of the reported taxa 84% were wild and only 16% were cultivated (Figure 2).

Of the 301 plants identified to species or genus, only 5 species were exotics (2%). They were: *Artocarpus altilis *(Parkinson) Fosberg, Moraceae; *Aloe vera *(L.) Burm. f., Asphodelaceae; *Plantago major *L., Plantaginaceae; *Cymbopogon citratus *(DC. ex Nees), Cyperaceae; *Ocimum basilicum *L., Lamiaceae [1,45,54,55,57-59].

### Plant parts used, ways of preparation and administration

Leaves were the most commonly used plant parts followed by roots, stem, bark, latex, fruit, caudex and rarely mentioned organs i.e. twigs, sap, seeds, tuberous rhizome, aerial roots (Table 3). The species were prepared mainly via decoction (56%) or used fresh (22%) (Table 4) while the most common way of administration was external (53%), followed by oral administration (45%) and application of drops in the eyes (3%). During the households interviews we registered a preserved remedy only in 1 household out of 16. This was an alcohol extract of Uña de gato (*Uncaria guianensis *(Aubl.) J. F. Gmel. or *Uncaria tomentosa *(Willd.) DC., Rubiaceae). None of the informants had western medicines at home.

**Table 3:** Plant parts used

Plant parts used	n	%
Leaves	408	47
Leaves and stem	105	12
Root	80	9
Stem	77	9
Bark	61	7
Latex	32	4
Leaves and bark	11	1
Leaves and root	11	1
Fruit	9	1
Top of caudex	9	1
Other	58	7

**Table 4:** Ways of preparation

Preparation	n	%
Decoction	512	56
Fresh	207	22
Steam bath	54	6
Emplast	47	5
Infusion	25	3
Cold water extract	23	2
Alcohol extract	21	2
Heated	15	2
Boiled emplast	10	1
Other	8	1

### Ailments treated

**Figure 3:**
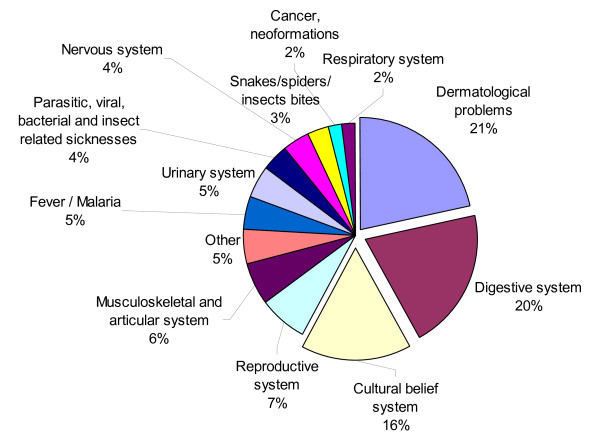
**Percentage of the medicinal use reports of the 402 medicinal plants to disease categories**. Dermatological problems, problems related to the digestive system and cultural belief system were among the most frequent ailments treated with the medicinal plants, representing 57% of all the medicinal use reports (n = 1268).

The 402 medicinal plants were used to cure in total 155 different ailments and diseases (Table 5). Problems related to the dermal system, digestive system and cultural belief system were among the most frequent ailments treated with the medicinal plants, representing 57% of all the medicinal applications (Figure 3).

**Table 5:** Ailments and diseases cured by the medicinal plant species collected in Bajo Quimiriki

Disease category	% of MUR	Specific diseases	No. of taxa	Medicinal Use Reports (MUR)
**Parasitic, viral, bacterial**	3.9	Chickenpox	3	3
**and insect related**		Cholera	4	4
**sicknesses**		Fungal infections	6	6
		Gonorrhoea	3	4
		Head lies	1	1
		Influenza	8	10
		Measles	2	2
		Rabies	1	1
		To prevent flu	1	3
		Uta (Leishmaniasis)	12	15
**Cancer, neoformations**	2.1	Cancer	4	4
		Hernia	4	4
		Prostate	12	15
		Stomach tumor	1	1
		To disinflamate cysts	1	1
		Tumors	1	1
**Dermic system**	21.8	'Arcoiris'	17	25
		'Pokio'	11	21
		Browses and swellings	20	39
		Burned skin	3	8
		Dandruff	2	2
		Haemorrhage	9	14
		Acne	6	7
		Skin rashes	1	1
		Skin rashes due to allergy	2	2
		Skin spots	11	23
		Sunburn	2	3
		To bathe babies	16	19
		To disinfect wounds	3	5
		To enhance beard growth	1	1
		To enhance hair growth	7	9
		To prevent formation of scars	1	1
		To prevent hair loss	12	25
		To prevent white hair	4	4
		To strenghten hair	1	1
		Warts	3	6
		Wound healing	21	53
		Wounds	4	7
**Digestive system**	20.3	Colics	6	15
		Diarrhoea	17	26
		Dysentery	2	2
		Emesis	5	5
		Gastritis	3	4
		Hepatitis	1	1
		Lack of appetite	1	1
		Liver-complaints	18	29
		Nausea	4	4
		Stomach ache	33	57
		Stomach acidity	9	14
		Stomach inflammation	3	3
		Stomach parasites	32	62
		To clean the stomach	2	2
		To extract caried teeth	2	3
		To make teeth fall	1	1
		To protect teeth	3	8
		To provoke emesis	1	1
		Toothpain	6	9
		Ulcers	6	10
**Musculoskeletal and**	6.1	Arthritis	1	1
**articular system**		Bone fractures	8	10
		Cramps	9	12
		Joint dislocations	7	8
		Osteoarthritis	13	19
		Pain in the muscles after work	2	3
		Scapular arthritis	4	6
		To relax the body	1	1
		Inflammation	3	3
		Internal inflammations	4	4
		Internal pain 'vaso'	3	3
		Pains in the body	7	7
**Nervous system**	3.9	Epilepsy	2	2
		Fatigue suppressor	2	2
		Headache	31	39
		Memory problems	1	1
		Relaxant	2	2
		Sleep disorders	1	1
		To make babies sleep	2	2
**Reproductive system**	7.2	To abort	10	22
		Disinflamation following parturition	2	2
		Galactagogue	1	1
		Menstruation pain	6	6
		Ovary infection	1	1
		Ovary inflammation	11	17
		Penis extender	3	4
		Sexual invigorator for men	6	9
		To correct irregular menstruation	2	3
		To enhance women fertility	4	5
		To give birth rapidly	3	3
		To give birth without pain	3	3
		To give birth rapidly and not feel the pain	3	3
		To lift up testicles	1	2
		Contraceptive	5	5
		To release the placenta after giving birth	1	2
		Vaginal infection	3	3
**Respiratory system**	1.9	Asthma	1	1
		Bronchitis	1	4
		Cold	3	6
		Cough	7	12
		Tubercolosis	1	1
**Snake/spider/**	3	Ant bites	1	1
**insect bites**		Insect bites	6	19
		Snake bites	13	16
		Spider bites	2	2
**Fever/Malaria**	4.9	Fever	16	33
		High Fever	1	3
		Malaria	10	26
**Urinary system**	4.8	Infection of urinary duct	5	7
		Inflammation of urinary duct	2	2
		Kidney-complaints	24	50
		For babies to stop wetting the bed	2	2
**Cultural belief system**	15.9	Chacho	14	23
		Mal agua	1	1
		Mal aire	51	82
		Seeing shadows	6	8
		To bathe babies	16	19
		To become adult	1	1
		To bring good luck	1	1
		To connect with the spirits of the forest	1	2
		To diagnose illnesses	1	2
		To get ones spirit back	2	2
		To make babies talk	1	1
		To make babies walk fast	8	9
		To protect from witchery and illness	2	4
		To see other places	1	2
		To see who is the responsible for a witchery	1	1
		To strenghten newborn babies	9	11
		Used by tobacco healer	1	6
		Witchery	20	27
**Other**	5	Against laziness in children	1	1
		Alcoholism	2	4
		Anemia	2	2
		Cholesterol	1	1
		Earache	3	3
		Eye infection	3	9
		Eye inflammation	4	4
		General not well being	1	1
		Swallen feet	4	8
		To attract men	2	2
		To attract the other sex	3	3
		To attract women	3	4
		To be faithful to the partner	1	1
		To boost immune system	1	1
		To gain weight	1	1
		To live long	1	1
		To loose weight	2	2
		To prevent ageing	5	8
		To stop dreaming dead people	1	1
		To strenghten elderly people	2	2
		To strenghten the body	3	3
		Violent men	1	1

The ailments and diseases are divided into disease categories with respective number of species.

During the household interviews, the diseases most frequently reported as having occurred in the families were (in decreasing order): 'mal aire', malaria, diarrhoea, 'chacho', headache and intestinal parasites (Table 6). Mal aire, a condition provoked by the accidental encounter with a spirit or by a cold wind, was cured in 88% of the cases with external treatments, either washing the body with a plant decoction or with a steam bath. Malaria was commonly referred to as 'paludismo' by the inhabitants of the community; its symptoms were described as high fever, tremors, headache, pallor, absence of appetite. All the informants indicated the cause as the bite of infected mosquitoes. The plants indicated to cure malaria were often called 'kepishiri' which means 'bitter' or 'shawetashi' or 'shawetapini' from the word 'shaweta' which is a local word for 'butterfly', probably locally related with bitterness. All the remedies against malaria were taken orally. Diarrhoea was very frequent and the informants attributed its cause to the excessive consumption of fruits or the mixing of sour and sweet food. Eighty-nine percent of the remedies against diarrhoea were taken orally. 'Chacho' was considered a dangerous disease that occurred quite often and frequently required the intervention of the 'curandero' or the shaman. The local inhabitants recognised two forms of chacho: 'chacho de cerro' and 'chacho de agua'. The first occurred when the forest of the hills did not permit trespassing, or by falling asleep on a special rock in the forest. Contrastingly the second was provoked by the malevolent influence of spirits which resided in the water, usually in the river. The symptoms were fever, vomit, headache and body pain. It was cured in 77% of the cases by washing with an herbal decoction. Headache was a common ailment; it was treated in 47% of the reports by pouring in the eye a drop of latex or an extract of the leaves, stem or roots of the medicinal plant. The other ways of administration against headache were washing the body in plant decoction (28%), oral administration (20%) and steam bath (5%). The cause of intestinal parasites was recognized as associated with consuming non-washed fruit and drinking non-boiled water. Intestinal parasites affected numerous adults and children. Ninety-four percent of the remedies were administered orally and six percent externally. One informant reported that the latex of *Artocarpus altilis *(Moraceae) should be applied on the belly bottom.

**Table 6:** Diseases most often occurring in the informant's families and the species used in their cure

Most mentioned diseases in the community	Percentage of informants reporting disease in their family	Species used to cure the disease in the community
Mal aire	62%	**Acanthaceae**: *Justicia appendiculata ***Apocynaceae**: *Himatanthus sucuuba ***Araceae**: *Anthurium dombeyanum*, *Philodendron plowmanii ***Arecaceae**:*Iriartea cf. deltoidea*. **Aspleniaceae**: *Asplenium *sp. **Asteraceae**: *Munnozia hastifolia, Tagetes erecta, Trixis divaricata ****Bignoniaceae: ****Mansoa alliacea ****Combretaceae: ****Terminalia *sp. **Costaceae**: *Costus *spp. **Cucurbitaceae**: *Momordica charantia ***Cyclanthaceae**: *Cyclanthus bipartitus, Carludovica palmata ***Euphorbiaceae**: *Ricinus communis ***Fabaceae**: *Desmodium *sp., *Inga *sp. **Gesneriaceae**: *Corytoplectus speciosus ***Lamiaceae**: *Ocimum basilicum ***Nyctaginaceae**: *Neea *sp. **Piperaceae**: *Piper aduncum*, *Piper cf. longestylosum, Piper *spp. **Rubiaceae**: *Psychotria poeppigiana ***Solanaceae**: *Brunfelsia grandiflora*, *Cestrum hediondinum, Nicotiana tabacum ***Urticaceae**: *Urera cf. baccifera, Urera cf. capitata, Urera laciniata ***Verbenaceae**: *Lippia alba ***Zingiberaceae**: *Costus *sp.
Malaria	38%	**Asteraceae**: *Bidens *sp.*, Clibadium sylvestre, Hebeclinum macrophyllum, Munnozia hastifolia ***Cucurbitaceae**: *Momordica charantia ***Solanaceae**: *Cestrum hediondiunum ***Verbenaceae**: *Phyla *sp.*, Verbena *sp.
Diarrhoea	31%	**Apocynaceae**: *Tabernaemontana *sp. **Asteraceae**: *Acmella oleracea*, *Bidens *sp., *Hebeclinum macrophyllum ***Bixaceae**: *Bixa orellana ***Chenopodiaceae**: *Chenopodium ambrosioides ***Cucurbitaceae**: *Momordica charantia ***Cyperaceae**: *Cyperus *sp. **Euphorbiaceae**: *Jatropha curcas ***Lauraceae**: *Aniba canelilla ***Menispermaceae**: *Chondrodendron tomentosum ***Myrtaceae**: *Psidium guajava ***Solanaceae**: *Physalis pubescens, Solanum americanum ***Verbenaceae**: *Verbena *sp.
Chacho	23%	**Asteraceae**: *Acmella oleracea, Eclipta prostrata, Mikania micrantha, Senecio *sp. *Tessaria integrifolia ***Cyperaceae**: *Eleocharis *sp. **Equisetaceae**: *Equisetum giganteum ***Fabaceae**: *Inga *sp. **Maranthaceae**: *Calathea *sp. **Onagraceae**: *Ludwigia peploides*
Headache	15%	**Anacardiaceae**: *Tapirira guianensis ***Araceae**: *Anthurium dombeyanum ***Asteraceae**: *Mikania micrantha ***Campanulaceae**: *Centropogon *sp. **Euphorbiaceae**: *Alchornea *sp. **Fabaceae***: Phaseolus *sp. **Piperaceae**: *Piper *sp. **Poaceae**: *Acroceras cf. zizanoides, Chusquea *sp., ***Rubiaceae: ****Hamelia patens, Psychotria poeppigiana ***Solanaceae**: *Cestrum hediondinum, Nicotiana tabacum, Solanum americanum, Solanum mammosum, Solanum *spp. **Urticaceae**: *Urera cf. Baccifera ***Verbenaceae***: Lantana camara*
Intestinal parasites	15%	**Amaranthaceae**: *Iresine diffusa ***Annonaceae**: *Annona muricata ***Apocynaceae**: *Himatanthus sucuuba ***Araceae**: *Anthurium dombeyanum, Anthurium kunthii, Homalonema crinipes, Philodendron brandtianum, Philodendron deflexum, Philodendron ernestii, Philodendron hylaeae, Philodendron plowmanii, Rhodospatha latifolia, Syngonium podophyllum ***Arecaceae**: *Bactris gasipaes ***Asteraceae**: *Ageratum conyzoides, Bidens *sp., *Hebeclinum macrophyllum ***Bignoniaceae**: *Tynanthus polyanthus ***Chenopodiaceae**: *Chenopodium ambrosioides ***Cucurbitaceae**: *Momordica charantia ***Elaeocarpaceae**: *Sloanea *sp. **Euphorbiaceae**: *Alchornea sp., Jatropha curcas ***Fabaceae**: *Inga *sp. **Melastomataceae**: *Miconia *sp. **Menispermaceae**: *Chondrodendron tomentosum ***Moraceae**: *Artocarpus altilis ***Solanaceae**: *Physalis pubescens*

### Plants used against Leishmaniasis

Leishmaniasis, an endoparasitic disease whose symptoms are skin ulcers, was locally known as 'uta' and the inhabitants distinguished two forms of it: 'uta seca' (dry uta) and 'uta de agua' (watery uta). Eleven species were reported against this disease (Table 7).

**Table 7:** Medicinal plants used in the treatment of Leishmaniasis

Species	Plant part used
*Anacardium occidentale*	Fruit pericarp oil
*Tapirira guianensis*	Bark
*Asclepias curassavica*	Latex
*Erechtites hieracifolius*	Leaves
*Jacaranda copaia*	Ashes
*Alchornea *sp.	Latex
*Croton lechleri*	Latex
*Jatropha curcas*	Latex
*Inga *sp.	Bark
*Psychotria poeppigiana*	Leaves
*Urera *cf. c*aracasana*	Sap

### Plants of cultural and social use

A special group of plants were the so called 'pinitsi' and 'ivenki': these seemed to be the most traditional and sometimes secret plants and were always planted near the house. The pinitsi were small herbs of which unfortunately none could be identified either because their owners did not allow their collection or because they asked that their identity and use would not be revealed to others. These were respectively the cases for the shaman and the local midwife. The ivenki (*Cyperus *spp.) were tall herbs often planted close to the entrance to the homegarden. The most frequent uses of the *pinitsi *and *ivenki *were to alleviate parturition pains, for children care (for example to bathe the babies to make them stronger against illnesses, to make babies sleep, to cure fever in children) and against sicknesses in the cultural belief.

Coca leaves (*Erythroxylum coca *Lam., Erythroxylaceae) were chewed together with the bark of a vine called 'chamairo' (*Mussatia hyacinthina *(Standl.) Sandwith, Bignoniaceae) and limestone as an alkaline additive. The chamairo bark sweetened the chew, making it more palatable. All the adults made use of this chew to avoid hunger and tiredness while working in the chacra or walking in the forest, but only one informant cultivated a bush of coca. Usually coca leaves, chamairo bark and limestone were bought from specialized stalls in Pichanaki.

### The traditional healers

During the interviews in the households four persons were generally mentioned as the most knowledgeable in the community regarding medicinal plants, they were three men and a woman: a shaman, a curandero, a tabaquero and a vaporadora. Each of them had specific competencies in the field of traditional healing.

The shaman had acquired his knowledge by visiting and paying other shamans, and he had started this apprenticeship in adult age. He was 53 years old, had a spouse but no children. By drinking a decoction of the vine 'Ayahuasca' (*Banisteriopsis caapi *(Spruce ex Griseb.) C.V. Morton, Malpighiaceae) he obtained visions which enabled him to diagnose diseases to his patients and resolve conflicts in the community. In this he was helped by the forest animals, whose eyes he could 'lend' by drinking the brew of Ayahuasca. Among his patients there were also many 'colonos', non-indigenous people, coming from as far as Lima. Maybe in order to attract even more customers, the shaman had included among his practices also fortune-telling.

The curandero was a 69 years old man, many villagers said that he had knowledge and powers like the shaman, but he was very discrete and did not confirm this when interviewed. Apparently he also received customers from the city.

The tabaquero was a man in his seventies, who did not speak Spanish, possibly by own choice. He was specialized in the healing with tobacco leaves performed by blowing smoke on the body of the ill patients.

The woman was a renowned vapour healer in her fifties. In the Asháninka communities, this healing practice is in the domain of skilled women who start their apprenticeship in an early age, following a strict diet. During the time of the fieldwork she became ill and was taken to another community to recover. Her family did not exclude that her illness could derive from the envy of one of the other healers.

A lady aged 71 proved to have an extensive knowledge of medicinal plants during the forest walk, although she was not mentioned as frequently as the four healers by the other villagers. She worked from time to time as a midwife, especially for women in the city.

### Knowledge variations

**Figure 4:**
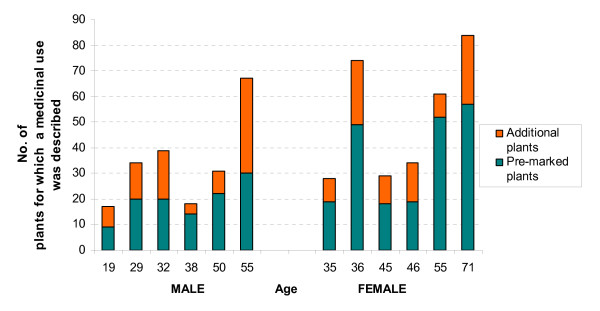
**Medicinal plants' knowledge variation during the forest walk**. Number of medicinal plants recognised by each informant on the track in the forest. Female informants identified and described the medicinal use of 49.5% of the pre-marked plants, while men did so for 26.6% of the pre-marked plants. All the informants indicated also a number of additional medicinal plants.

Eight hundred and sixty four independent events (72 pre-marked plants × 12 informants) were recorded during the forest walks in the community reserve, with six informants for each gender. An 'event' is here defined as "the process of asking one informant on one day about the uses they know for one species" [17]. All the informants, with varying extent, spontaneously pointed out also at plants that were not pre-marked and described their medicinal application. The number of these plants is 91 for men and 96 for women. The results from the forest walks are shown in Figure 4. Women described a medicinal application in a higher number of events: they scored a total of 310 record of use versus 206 total records of use by men. The difference is mainly given by the 72 pre-marked plants of which women described one or more medicinal use in 49.5% of the events, while men did so in 26.6% of the events. The difference is statistically significant (χ^2 ^= 47.1429, p-value = 6.6e-12). In the group of men the oldest informant (55 years old) was the most knowledgeable, indicating a medicinal use for 30 out of the 72 pre-marked medicinal plants; also in the group of women the most knowledgeable informant was the oldest person (71 years old), who indicated a medicinal use for 57 of the 72 pre-marked medicinal plants. In the group of women two other informants aged 36 and 55 were particularly knowledgeable indicating a medicinal use for 49 and 52 of the 72 pre-marked medicinal plants each. Age explains 46% of the variation of knowledge between the 12 informants. The correlation of the variables is statistically significant, with a 95 percent level of confidence (p-value = 0.015). Nevertheless if we consider the group of men and women separately, we see that for men age explaines 68% of the variation and the correlation between the variables is still statistically significant at 95 percent level of confidence (p-value = 0.042), while for women age explains only 35% of the variation in the group and the correlation between age and knowledge is not statistically significant (see Discussion).

We registered a great variation of uses attributed to the same species by different informants, even for those plants that were well known in the community (whose medicinal use was reported by more than 10 informants). As an example *Dieffenbachia costata *Klotzsch ex Schott (Araceae) was reported by 23 informants against 10 different conditions/diseases (see Table 8 and Additional file 1).

**Table 8:** Medicinal plants reported by at least 10 informants and their respective uses

Scientific name (Voucher N.)	No. of Informants	Medicinal uses
*Dieffenbachia costata *(0704, 0605, 2813, R38, R77)	**23**	Insect bites, Bone pain, Bronchitis, Cold, Cough, Snake bites, Wounds, To protect from sorcery and illness, Influenza, Toothache.
*Uncaria guianensis *(R19, PA3, R56, 4015, 4017)	**19**	To abort, Osteoarthritis, Cancer, Cold, Inflammation, Kidney-complaints, Liver-complaints, Ovary inflammation, Penis extender, Prostate, Stomach ache, Ulcers.
*Cestrum hediondinum *(0607, 2904, R91)	**17**	Fever, Headache, Malaria, Stomach ache, Sorcery.
*Homalomena crinipes *(R4, R50)	**15**	To clean the stomach, Toothache, To Abort, To protect teeth, Intestinal parasites.
*Acmella oleracea *(0203, PL22)	**15**	Sunburn, 'Pokio', Browses and swellings, 'Chacho', Diarrhoea, Insect bites, Toothache, Earache, 'Arcoiris'.
*Hebeclinum macrophyllum *(R33, 0107, R7)	**14**	Malaria, Stomach ache, Infection of urinary duct, Liver-complaints, Intestinal parasites, Chickenpox, Diarrhoea, Stomach acidity, Colics.
*Erechtites hieraciifolius *(0211, PL23)	**14**	Skin spots, Acne, Uta (Leishmaniasis), To prevent hair loss.
*Chenopodium ambrosioides *(PL1, 0206, 0602, 0907)	**14**	Intestinal parasites, Colics, Fever, Diarrhoea, To bathe babies.
*Piper aduncum *(R10, 0603, 2806)	**14**	Cramps, High Fever, 'Mal aire', Menstruation pain, Stomach ache, Ulcers, Vaginal infections, Vomit, Sorcery, Wound-healing.
*Xanthosoma poeppigii *(R89)	**13**	Wound healing, Bone fractures.
*Momordica charantia *(0108, 2901)	**13**	Malaria, Stomach ache, Stomach acidity, Fever, Diarrhoea, Intestinal parasites, Kidney-complaints, Mal aire.
*Verbena *sp. (2920, 3701)	**13**	Malaria, Stomach ache, Diarrhoea, To bathe babies.
*Syngonium podophyllum *(R9, 2814, DH09, DH04)	**12**	To correct irregular menstruation, To extract caried teeth, Toothache, Contraceptive, Intestinal parasites, Joint dislocations, Anaemia, Osteoarthritis, Browses and swellings.
*Munnozia hastifolia *(R27)	**12**	Cholera, Sorcery, Kidney-complaints, Gastritis, Wounds, Malaria, To stop dreaming dead people, Stomach ache, 'Mal aire', Fever, Cholesterol.
*Croton lechleri *(0609)	**12**	Wound healing, Ulcers, Liver-complaints, Uta (Leishmaniasis), Dysentery, Disinflamation following parturition.
*Chaptalia nutans *(2811, 2908, R34)	**11**	Malaria, Stomach ache, Diarrhoea, Intestinal parasites.
*Bixa orellana *(2810, 2907)	**11**	To prevent hair loss, Eye infection, Prostate, Diarrhoea, Wounds, Stomach inflammation, Cancer, Kidney-complaints, To bathe babies.
*Xiphidium caeruleum *(R55,0606, R41, 4004, 4007)	**11**	Bone fractures, Joint dislocations, Browses and swellings, To strenghten hair, To enhance hair growth, 'Mal agua', To bathe babies.
*Hamelia patens *(2803, 2903)	**11**	To disinfect wounds, Browses and swellings, Stomach ache, Headache.
*Urera cf. Baccifera (R90, R92)*	**11**	Mal aire', Kidney-complaints, Seeing shadows, Headache, Pain in the muscles after work, General not well being, Cramps.
*Asclepias curassavica *(R29)	**10**	Wound healing, Uta (Leishmaniasis), Eye inflammation.
*Anthurium dombeyanum *(R23, R64)	**10**	Snake bites, 'Mal aire', To attract men, Headache, To enhance hair growth, Intestinal parasites, Infection of urinary duct, Stomach ache, Cough, Seeing shadows.
*Bidens pilosa *(0207, R3, LI25, PL13, ROM1)	**10**	Burned skin, To prevent formation of scars, To give birth rapidly, To prevent hair loss, Dandruff, Contraception, To make babies walk fast, Skin spots.
*Mikania micrantha *(PL18)	**10**	Headache, Osteoarthritis, Scapular arthritis, 'Chacho', Sorcery, Arcoiris, 'Pokio', Alcoholism, Acne.
*Tessaria integrifolia *(0102, PL21)	**10**	Kidney-complaints, Ovary inflammation, General not well being, Swollen feet, 'Pokio', 'Chacho'.
*Nicotiana tabacum *(0601)	**10**	Insect bites, Used by tobacco healer to cure patients, 'Mal aire', Headache.
*Urera laciniata *(R32, 2817)	**10**	Sorcery, Kidney-complaints, Prostate, Cramps, Sorcery, 'Mal aire', Osteoarthritis, Fever, Measles, Improve male libido.

### Medicinal plants known by the children

The children of the 5^th ^and 6^th ^grade of the elementary school stated that the persons that knew most about medicinal plants in their families were the grandfathers. Out of seventeen children in the class, two (a boy and a girl) declared that they would have liked to become a shaman. The class listed 27 medicinal plants with their respective uses and preparations, which are displayed in Table 9. The children knew the great majority of plants with a Spanish name, only three plants were called with an Asháninka name. They were able to describe the way of preparation of 14 of the 27 plants. Twenty plants were commonly growing in or around the homegardens and ten of these were actively cultivated. The botanical names of the table have been inferred from the vernacular name and therefore in some cases more than one species might correspond to a vernacular name.

**Table 9:** Medicinal plants known by the children of the 5th and 6th grade of the elementary school

	Vernacular name	Scientific name	Family	Use	Preparation*
	Pusanga	‒	‒	To attract the other sex	‒
✦	Chupa sangre	*Hamelia patens*	Rubiaceae	Wounds, colics	‒
	Cuatro esquinas	‒	‒	Browses	‒
❧✦	Verbena	*Verbena *sp.	Verbenaceae	Malaria	‒
❧✦	Matico	*Piper aduncum*, *Piper *sp.	Piperaceae	Wounds, fever	Bw
✦	Amargón	*Hebeclinum macrophyllum*	Asteraceae	Colics, diarrhoea	Bw, Bd
✦	Suelda suelda	*Cissus gongylodes*, *Commelina *sp.	Vitaceae	Bone pain	Em
✦	Paico	*Chenopodium ambrosoides*	Chenopodiaceae	Belly/Stomach ache, colics, diarrhoea	‒
	Oja de Murcielago	*Munnozia hastifolia*	Asteraceae	Ovary inflammation, belly ache	Bd
✦	Llantén	*Plantago major*	Plantaginaceae	Ovary inflammation, belly ache	‒
❧✦	Sangre de grado	*Croton lechleri*	Euphorbiaceae	Wounds	Lx
	Sachahuasca	‒	‒	Kidney-complaints	Bd
	Uña de gato	*Uncaria guianensis Uncaria tomentosa*	Rubiaceae	Wounds, kidney-complaints	Bd
❧✦	Pan de arbol	*Artocarpus altilis*	Moraceae	Hernia	Lx
	Huampo Blanco	*Heliocarpus americanus*	Tiliaceae	To purify the body	Bd
❧✦	Platano	*Musa paradisiaca*	Musaceae	Wounds, haemorrhage	Sa
❧✦	Pepa de palta	*Persea americana*	Lauraceae	Diarrhoea	Cbd
	'Okilia'	‒	‒	Eye infection	Cs
❧✦	Achiote	*Bixa orellana*	Bixaceae	'Susto', eye infection	‒
✦	'Porokishi'	*Vernonia *sp.	Asteraceae	Wounds	‒
❧✦	Tabaco	*Nicotiana *sp.	Solanaceae	'Susto', sorcery, used by curanderos	‒
❧✦	Piñon	*Jatropha curcas*	Euphorbiaceae	Parasites	‒
❧✦	Guajaba	*Psidium guajava*	Myrtaceae	Diarrhoea, stomach ache	Bd
✦	Chanca piedra	*Phyllanthus niruri Phyllanthus orbiculatus*	Euphorbiaceae	Wounds, malaria	‒
✦	Lengua de perro	*Chaptalia nutans*, *Asplenium serratum*	Asteraceae	Stomach ache, ovary inflammation	‒
✦	Cola de caballo	*Xiphidium caeruleum*	Haemodoraceae	'Mal aire', sorcery, to enhance hair growth	St, Bw
✦	Pajaro bobo	*Tessaria integrifolia*	Asteraceae	Head ache	‒

	❧: cultivated in homegardens				
	✦: grows wild in homegardens				
	*Bw: boil and wash with the decoction; Bd: boil and drink the decoction; Em: emplast; Lx: apply the fresh latex on the wound; Sa: apply sap on the wound; Cbd: crush the seed, boil and drink; Cs:crush and squeeze into the eye; St: steam bath				

### Comparison with the Yanesha ethnic group

A recent study published on the medicinal concepts and plants uses among the Yanesha group [60] allows a comparison with the Asháninka of our study. The data on the Yanesha were collected in three communities in the Oxapampa province (Pasco Region) over a period of three years, working with 30 informants. Six hundred and seven herbarium samples were collected all together, and a total of two hundred and forty nine species were designated as medicinal [60]. Our data on the Asháninka were collected over a period of three months in one community working with a total of 37 adult indigenous informants and 17 children. In this short period we collected a total of 402 herbarium samples corresponding to at least two hundred and eight different species (Additional file 1).

Comparing the two sets of data we found an overlap of 33 species (16% of our 208 identified species). In 64% of the cases the plants were used for the same disease or condition (see Table 10).

**Table 10:** Comparison between medicinal use of the same species by the Asháninka of Bajo Quimiriki and the neighbouring group Yanesha, based on data from the present study and Valadeau *et al*. 2010 [60]

Botanic family *Scientific determination *(herbarium number)	Use by the Yanesha (from Valadeau *et al*. 2010)	Use in the Asháninka community of Bajo Quimiriki
**Amaranthaceae**		
*Iresine diffusa *Humb. & Bonpl. ex Willd. (CA06, IS02, LI4, PE2)	Malaria	Intestinal parasites, liver complaints
**Araceae**		
*Syngonium podophyllum *Schott (R9, 2814, DH09, DH04)	**Wounds**, burns	To correct irregular menstruation, to extract caried teeth, toothache, contraceptive, Intestinal parasites, joint dislocations, anaemia, osteoarthritis, **browses and swellings**
*Philodendron ernestii *Engl. (FL25)	**Intestinal parasites**	**Intestinal parasites**
**Arecaceae**		
*Bactris gasipaes *Kunth (DH05)	Mal aire	Intestinal parasites
**Asteraceae**		
*Bidens pilosa *L. (0207, R3, LI25, PL13, ROM1, FL3)	Flu, muscular pain, liver pain, fever, malaria, urinary and uterine infections, trauma, to have an eternal love	Burned skin, to prevent formation of scars, to give birth rapidly, to prevent hair loss, dandruff, contraceptive, to make babies walk fast, skin spots
*Mikania micrantha *Kunth (PL18)	Mal aire, **protection against the illnesses from the river**, throat pain	Headache, arthritis, **chacho**, sorcery, **arcoiris, pokio**, alcoholism, acne
*Munnozia hastifolia *(Poepp.) H.Rob. & Brettell (R27)	General body pain, **stomach ache**, gynaecological disorder, **kidney pain**, leishmaniasis, infected **wounds**, to clean the baby just after birth, **stomach infection**, diarrhoea, liver pain, protection for children	Cholera, sorcery, **kidney-complaints**, **gastritis**, **wounds**, malaria, to stop dreaming dead people, **stomach ache**, mal aire, fever, cholesterol
*Tessaria integrifolia *Ruiz & Pav. (0102, PL21)	Liver and **kidneys pain**, urinary inflammation, prostatic pain, throat pain	**Kidney-complaints**, ovary inflammation, general not well being, swallen feet, 'pokio', 'chacho'
**Bignoniaceae**		
*Jacaranda copaia *D.Don (R93)	Malaria or fever preventive, infected pimples, itching dermatosis, **leishmaniasis**	**Leishmaniasis**, wound healing
*Mansoa alliacea *(Lam.) A.H. Gentry (4022)	**Diarrhoea with stomach pain, fever**, flu, cold, rheumatic pain, skin ulcers, boils	**Mal aire**
**Bixaceae**		
*Bixa orellana *L. (2810, 2907)	Heart pain, **prostate inflammation**, anorexia, **eye pain**	To prevent hair loss, **eye infection**, **prostate**, diarrhoea, wounds, stomach infection, cancer, kidney-complaints, to bathe babies
**Chenopodiaceae**		
*Chenopodium ambrosioides *L. (PL1, 0206, 0602, 0907)	**Abdominal pain with gas**, **intestinal parasites**	**Intestinal parasites**, **colics**, fever, diarrhoea, to bathe babies
**Cyclanthaceae**		
*Cyclanthus bipartitus *Poit. (R87)	Ant's bite (fever)	To prevent hair loss, sorcery, mal aire
**Euphorbiaceae**		
*Euphorbia heterophylla *L. (WA01, 2915)	**Leishmaniasis**	**Wound healing**, eye infection
*Manihot esculenta *Crantz (2914)	Mal aire: muscular pain	To cure bites of dogs with rabies
**Fabaceae**		
*Calliandra angustifolia *Spruce ex Benth. (1002, 0604)	**Tiredness of old persons**, stomach ache, purgative	To strenghten newborn babies, to make babies walk fast, **to strenghten elderly people**
**Gleicheniaceae**		
*Dicranopteris pectinata *(Willd.) Underw. (PE29)	General malaise	To strenghten newborn babies
**Haemodoraceae**		
*Xiphidium caeruleum *Aubl. (R55,0606, R41, 4004, 4007)	**Hair care**, **wounds**	Bone fractures, joint dislocations, **browses and swellings**, **to strenghten hair**, **to enhance hair growth**, 'mal agua' to bathe babies
**Iridaceae**		
*Eleutherine bulbosa *(Mill.) Urb. (2103, 2909)	Intestinal **haemorrhage**, **vomiting with blood**, fever	Contraceptive, **haemorrhage**
**Malvaceae**		
*Gossypium barbadense *L. (0508, 0906)	Ear pain, caterpillar burn, dysentery, abdominal cramps, post partum care	To strenghten newborn babies, acne
*Sida rhombifolia *L. (WA02, 2924)	Swellings, gynaecological disorders, pain in the ovary, rheumatic pain, arthritis, heart pain	To prevent hair loss, colics, mal aire, kidney-complaints
**Myrtaceae**		
*Psidium guajava *L. (2930)	**Diarrhoea **with intestinal cramps and fever	**Diarrhoea**
**Piperaceae**		
*Piper peltatum *(L.) Miq. (2911, 0114, 4003)	Boils, venomous fish bite, wounds, infection **post partum**	To give birth without pain, **to release the placenta after giving birth**
*Piper aduncum *L. (R10,0603, 2806)	Boils, **fever**, rheumatism, cold	Wound healing, **high fever**, ulcers, mal aire, vaginal infection, sorcery, menstruation pain, cramps, emesis stomach ache, cough, to disinfect wounds, fungal infections
**Rubiaceae**		
*Hamelia patens *Jacq. (2803, 2903)	Threath of miscarriage, fever, malaria, dysentery, burns, herpes, **skin infection**, disease caused by rainbow spirits	**To disinfect wounds**, browses and swellings, stomach ache, headache
*Uncaria guianensis *(Aubl.) J. F. Gmel. (R19, PA3, R56, 4015, 4017)	**Gynaecological disorder **(uterine haemorrhage), internal wounds, rheumatic pain, general fortificant	Osteoarthritis, kidney-complaints, ulcers, liver-complaints, cold, cancer, penis extender,**ovary inflammation**, prostate, to abort, stomach ache, inflammation
*Uncaria tomentosa *(Willd.) DC. (PA4)	**Gynaecological disorder **(uterine haemorrhage), internal wounds, **rheumatic pain**, general fortificant	**Arthritis**, kidney-complaints, stomach ache, inflammation, cold, prostate
**Scrophulariaceae**		
*Scoparia dulcis *L. (0912)	Burns, herpes, skin infections, pimples (diseases caused by the rainbow spirits); 'susto': miscarriage caused by rainbow spirits, fever, dysenteria	To prevent hair loss
**Solanaceae**		
*Physalis pubescens *L. (WA5)	Ovary pain	Stomach ache, diarrhoea, intestinal parasites
**Tiliaceae**		
*Heliocarpus americanus *L. (R14)	**Stomach ache **with strong fever, **kidney pain**, traumas, bruises, swellings, to facilitate birth	Gastritis, **stomach ache**, prostate, to give birth rapidly, **kidney-complaints**, ovary inflammation, inflammation of urinary duct
**Urticaceae**		
*Urera cf. baccifera *(R90, R92)	**Diarrhoea, stomach ache, rheumatic pain, cough, flu**	**Mal aire**, kidney-complaints, seeing shadows, headache, pain in the muscles after work, **general not well being**, cramps
*Urera laciniata *Wedd. (R32, 2817)	Malaria	Sorcery, kidney-complaints, prostate, cramps, Sorcery, mal aire, osteoarthritis, fever, measles, to improve male libido
**Verbenaceae**		
*Stachytarpheta cayennensis *(Rich.) Vahl (2805)	**Snake bites**	**Snake bites**

Ninety seven percent of the specific uses reported by the Yanesha were also reported by the Asháninka, including particular diseases like 'arcoiris' (skin problems occurring when bathing in the river under a rainbow). The forms of preparation of the remedies were also very alike, including among others steam baths or application of leaf sap in the eyes. In both ethnic groups plants from the genus *Piper *(Piperaceae) were very often prepared in the form of a steam bath against 'mal aire'.

## Discussion

### Medicinal plants - diversity, life form and habitat

The families Asteraceae, Araceae, Rubiaceae, Euphorbiaceae, Solanaceae and Piperaceae, were the most important families in terms of number of taxa with medicinal uses in the community. A recent study among the Yanesha of the Selva Central reported similar results, with exception of the Asteraceae which was represented in their pharmacopoeia with only seven species [60]. According to Vásquez Martínez *et al*. [61] these families are also the most commonly encountered in the Peruvian Selva Central. At the same time these families are often targeted during taxonomic approaches to drug discovery for their rich content of secondary compounds like steroids and alkaloids [46] and many of their species are well-known South American medicinal plants [49,50]. The prevalence of Asteraceae among local pharmacopoeias is reported in many other South American studies [25,27,62,63]. The Araceae family was of particular importance in the community: 17 species were indicated for medical use by the informants, while the previously mentioned study among the Yanesha [60] recorded the medicinal use of ten species in this family. The Araceae, especially those in the genus *Philodendron*, were often called 'kainto': the same indigenous name was also reported in studies among the Ashéninka of the Ucayali and Ashéninka del Gran Pajonal [29]. The results from the comparison with the Yanesha showed that the two ethnic groups shared at least 16% of the reported pharmacopoeia and they had in common the great majority of their diseases and traditional healing practices. The fact that none of the names were in common suggests that the communities independently discovered these plants.

In ethnobotanical studies the prevalent life-form of medicinal plants varies between herbs [64,65] and trees [66], with leaves and stems as the most commonly used plant parts [25,64,66,67], this is in agreement with the observations in the Bajo Quimiriki community. Using leaves is less destructive than bark stripping or digging out the roots.

Only 5 of the medicinal plants identified to species or genus (301) were exotics (2%), while other studies reported percentages of exotic plants among the local pharmacopoeias as high as 37.5% in Loja and Zamora-Chinchipe (Ecuador) [20] and 26.5% in Rama midwifery (Nicaragua) [68]. This indicates that the Asháninka in this community have a strong tradition of medicinal plants and that an eventual plant exchange between communities involves mainly local species. The families of the plants identified only to family level are all common in Peru. The forty-seven plants which were not identified were collected for the great majority in the forest and are therefore very likely to be native species, as all the plant species identified as exotics were found in the homegardens and were well known introduces species.

Our fieldwork was limited to 3 months in the area, therefore we probably missed some annual plants that were not found during our stay. The short fieldwork also decreased our possibilities to collect fertile voucher specimens and made the identification of many plants challenging.

### Plants of cultural and social use

Regarding the plants locally called "ivenki" and "pinitsi", the use of ivenkis among the Asháninka and Ashéninka has been reported previously [4,29,69], where ivenkis (*Cyperus *spp.) for each of the following categories have been indicated: hunting, war, physical illnesses, spiritual illnesses, pregnancy and parturition, children protection, agriculture, handworks, vanity [69]. The Matsigenka cultivate various sedges (Cyperaceae species) with medicinal properties, which they call 'ivenkiki': these are used as hunting medicine and for fertility control [70]. Also the Yanesha cultivate various species of sedges which they call "Piripiri" or "Epe'. Like the Asháninka, they cultivate these plants of high cultural significance around the houses and they use them in a wide array of situations, ranging from behaviour regulation to galactagogue [60]. Plowman *et al*. [71] reported that the efficacy of the medicinal plants from the genus *Cyperus *might be related to a rhizome infestation by the ascomycetous fungus *Balansia cyperi *(Clavicipitaceae) producing ergot alkaloids.

Information on pinitsis is scarce, probably as their use is often related to magical beliefs like love, enchantment and sorcery, which the users might want to keep secret. Pinitsis were also the only plants that the community did not permit to be collected and for which the shaman and the midwife requested that secrecy was maintained.

The habit of chewing dried coca leaves together with the bark of 'chamairo' is a custom already reported for the Asháninka living on the Apurimac and Ene rivers of Peru, the Amuesha of the river Pichis and was also reported among other indigenous tribes of eastern Bolivia, who used the crude ash of the spathe or leaf base of the *motacú *palm (*Scheelea princeps *(Mart.) Karst.) as an alkaline additive [72].

No specimens were collected in the community for the coca and chamairo plants, as the dried leaves and the stripped bark respectively were normally bought from stalls in Pichanaki. Nevertheless, from the observed reddish colour of the chamairo bark it can be deduced that it was the 'chamairo colorado' or 'red chamairo' identified as *Mussatia hyacinthina *(Bignoniaceae)[72].

### Ailments treated

The most frequent categories of use of the medicinal plants in the community of Bajo Quimiriki were: dermatological problems, digestive system and cultural belief system. These categories were also reported as the most important in studies carried out among indigenous people in Australia, Fiji, Haiti, India, Kenya, Mexico, Nepal, Nicaragua, North America, Peru, Rotuma, Saudi Arabia, Thailand, Tonga and West Africa [73]. This means that the categories of ailments treated in the community of Bajo Quimiriki with medicinal plants are the same suffered by the majority of rural people in developing countries, while they are different from the ailments categories treated by conventional drugs in developed countries. In the United States, for example, drugs are mainly used against microbial infections, nervous system affections, dermatology and cardiovascular diseases [73].

Problems affecting the skin include among others skin rashes, sunburn, 'arcoiris', 'pokio', hair care, wounds and haemorrhage. This category is also reported as the most important among the Yanesha [60]. Problems related to the digestive system are probably caused by the lack of sewage system and eggs of intestinal parasites present in water or non-washed fruit. Cultural belief system diseases occupy always a big percentage of the medicinal applications in indigenous groups. In the community of Bajo Quimiriki the most mentioned diseases in this category were 'chacho', 'mal aire', 'to bathe babies' and 'sorcery'. Asháninka women bathed their babies in herbal decoctions in order to protect them from illnesses and sorcery, to make them sleep, to calm them, to make them stop crying, to make them obedient, to make them grow healthy and with appetite, to make them sleep, to make them learn to walk fast. This last application indicates the wish of the women who need to go and work in the family *chacra *to have their children walking as soon as possible. Some of these uses were mentioned also by the Yanesha and reported in Valadeau *et al*. [60]. Among the diseases most often occurring in the informant's families (Table 6), malaria and 'chacho' were those for which the minor number of medicinal plants was reported. While chacho, a condition provoked by malevolent spirits, is often cured through the intervention of the shaman, malaria is more often cured at home and only in acute cases at the hospital. The scarcity of medicinal plants known to cure malaria might depend from the fact that this disease was introduced into South America from Africa during colonial times, and that therefore indigenous people do not have a long tradition of herbal remedies for it. However, studies in other South American indigenous communities have proven the contrary, reporting numerous species used for this disease [14,74]. The explanation could lie in a combination of the following factors: 1) there is a real scarcity of plants for curing malaria in the area, 2) malaria is treated with species that are used against fever, 3) loss of traditional knowledge, 4) preference to be treated in the hospital. All the informants were aware that malaria was transmitted by the bite of infected mosquitoes and mosquito nets were widely used. This is probably due to the fact that this disease and its prevention have been amply publicized by campaigns organized by the Peruvian Ministry of Health, which also distributed free mosquito nets in indigenous communities [75].

In contrast, the parasitic nature of leishmaniasis and the role of sand flies (family Psychodidae) in its transmission were not known by any of the informants and the medicinal plants employed were only applied topically on the visible wounds which are symptoms of the parasitic infection. This has been reported among different ethnic groups in the Loreto and Pasco departments of Peru [14,74]. The Yanesha of the Pasco department use the same spanish names 'uta seca' and 'uta de agua' to indicate the two kinds of leishmaniasis, but differently from the Asháninka of Bajo Quimiriki, they have a myth to explain the disease: an old man called *Mareñets *who, transformed into a fly, introduces himself into his victim's bodies through a bite and kills them from the inside to steal their spirit [74]. The lack of myths to explain this disease in the community of Bajo Quimiriki does not necessarily indicate acculturation and loss of traditions and believes: the Yanesha myth could also be a reinterpretation in a mythical frame of information from biomedical origin. Only one of the species mentioned against Leishmaniasis was in common with the Yanesha: *Jacaranda copaia *D.Don (Bignoniaceae). The way of preparation was different: while the Yanesha applied a poultice of boiled leaves on the affected area [74], our informants applied the ashes of the bark on the wounds. Extracts of the leaves of this tree have shown some leishmanicidal properties, but the products were found to be toxic for macrophages [76]. It would be interesting to test whether the use of ashes enhances the leishmanicidal activity, while reducing the toxicity previously reported in leaves extracts.

The gender distribution of our adult informants during the household interviews (11 women and 5 men) might have influenced our results on number of preserved remedies kept in the house. We registered an alcohol extract of 'Uña de gato' (*Uncaria guianensis *or *Uncaria tomentosa*, Rubiaceae) in only one of the 16 visited households. Men were often the ones preparing alcohol extracts in the house and it might be that in their absence their wife did not show these remedies to us.

### The shifting role of the traditional healers

Five people in the community were specialized in different forms of traditional healing. The three men indicated as the 'shaman', the 'curandero', and the 'tobacco healer' were in fact seemingly shamans. In this matter appearances can be deceptive. The 'curandero''s discretion is very typical as a really great shaman tends to minimize his powers, unlike the 'official' shaman, who proclaims himself so. And the 'tabaquero' belongs to a former stage of Ashaninka shamanism, before the arrival of Ayahuasca: 'sheripiari' (the Asháninka work for 'shaman') means 'tobacco drinker'. The man who in the community proclaimed himself shaman combined the use of medicinal plants with forms of fortune-telling typically widespread in the cities (cards reading, dice) not common to the community, while the other four specialists used only plants. Fortune-telling is probably to the shaman a way to satisfy the requests of customers coming from cities, a symptom of a shift in shamanism from a traditional practice to a source of income. The demand for services of the local specialists by the city inhabitants could be explained by the fact that many people in Pichanaki have rural origins and trust traditional knowledge. A second reason could be that many people in the city are poor and cannot afford to go to the hospital to give birth or to buy prescribed medicine. This was also mentioned by the local midwife as a reason why she was often called to assist women giving birth in the city.

### Knowledge variations

Traditional medicine was not only in the hands of the specialists: in every household visited some medicinal plants were cultivated or collected from the wild. Common ailments were cured by villagers themselves often using herbal remedies. Men knew plants that were used to treat women ailments and women knew plants used by men. This might be due to the fact that there was not a tradition of keeping dried plant material at home, therefore, when needed men or women would go to collect the herb(s) needed by their spouse or children. An exception was the practice of steam bathing, which seemed to be exclusive dominion of women. This is in accordance with other studies performed among Ashéninka in the Peruvian Amazons [77]. It should be stressed that knowledge was not always transmitted from parents to children. Some informants in their thirties lamented the fact that their parents did not want to teach them what they knew on medicinal plants.

The results of the forest walks indicated that there was a significant correlation between age of the informants and knowledge of medicinal plants. This result has been widely reported [18,32,78] and underlines the vulnerability of traditional plant medicinal knowledge if its transmission through the generations is limited by acculturation or inter-ethnic exchange. Lenaerts [22] reported indeed a loss of the Ashéninka distinctive knowledge among modern Asháninka who were borrowing traditional knowledge from Shipibo neighbours. Nevertheless, in our study the correlation was statistically significant if the whole group of informants was considered, while it was not significant if only the group of women was analyzed. In order to explain the performances of the informants we should consider their personal backgrounds. Flora, the informant aged 71 who had the greatest plant knowledge, was a midwife with a marked personal interest towards medicinal plants. Occasionally she also sold medicinal plants to colonos. Lidia, the exceptionally knowledgeable young female informant (36 years old), was the elder daughter of the local vapour healer and she said to have learnt much from her mother. She explained that when she was very young she wanted to become vaporadora too, but during adolescence she gave up the apprenticeship due to the rigid diet to respect. So, the personal background of this informant explains her particularly profound knowledge compared to her young age. This is also true for one of the main informants with whom the track was established and who provided information on almost all the pre-marked plants. He was 27 years old, brother of the actual leader of the community and son of the local 'curandero'. He said that he was very interested in the use of medicinal plants and that in the future he might have considered becoming a curandero himself. So, although there is a general trend of acquiring medicinal knowledge through the age, much of the variation between informants can be explained by personal interests and also by the relation with a local healer.

The high number of different uses reported for the same species by different informants could be a sign of regional homogenization through borrowing of ethnobotanical knowledge from other ethnic groups or neighbouring communities [22] or an indication of an ongoing experimentation with plants properties and lack of communication between the inhabitants who might want to keep their remedies secret to others. In our study the ethnobotanical information on forest plants were collected in a no-random way, using forest walks rather than transects or quadrants. The implications for results were that some of the plants remained unidentified and for some of them we collected ethnobotanical information only from one informant (although we tried to get additional information for 80 plants in the cross-check).

### Knowledge of medicinal plants among the children

The ethnobotanical information given by the children on 27 medicinal plants was consistent with the information provided by adults on the same plants. The children could not describe the preparation of half of the remedies, probably because at this age they have never actively prepared remedies, but only observed their parents or grandparents getting it ready. In a study on medicinal plant knowledge among mothers and children in Kenya, it was found that school children aged 13-16 participated actively in health care, treating both themselves and their younger siblings with medicinal plants [79]. Maybe this will be the case in the community of Bajo Quimiriki, where after the 6^th ^year of elementary school many of the children will discontinue schooling because their parents cannot afford the school fees.

Twelve out of the twenty-seven plants indicated by the children (44%) were among the most well-known plants in the community shown in Table 9 and 25 out of 27 plants grew in and around the homegardens. This shows that children's knowledge of medicinal plants is limited to the plants that grow around the households, which probably are used more often than those growing in the forest as they are more accessible. The children did not all have the same knowledge on medicinal plants, some of them were more active in giving names of medicinal plants while some others only confirmed what others had just said. This could be also influenced by shyness, but the oral assessment that we used did not allow us to clearly distinguish these differences. An individual written assessment combined with socio-economic data on the family of origin (Asháninka, colonos or mixed) would better explain how knowledge is transmitted to children.

### Comparison with the Yanesha

We found that 33 of our species (16% of the plants identified to species) were also used medicinally by the Yanesha. If our plants could have been all identified to species level we might have found more species in common between the two pharmacopoeias. In fact there were also 23 additional plants pertaining to the same genus in common which might have been actually the same species. Our fieldwork of three months in the area limited the possibility of finding fertile voucher specimens and therefore the identification presented some challenges. Nevertheless the fact that in only three months we were able to collect 402 voucher specimens indicates that there is an abundant knowledge and daily use of medicinal plants in this community.

The percentage of species with common uses among the two groups could also be higher if we for example considered that 'liver complaints' could be a symptom of 'malaria' or if we related 'anorexia' with 'stomach infection' and therefore counted them as an identical use. There are many concepts in common between the cosmology of the Yanesha and the Asháninka, like the belief in malignant spirits which reside in the mountains, in the lakes or in the rainbow, wandering souls which can enter ones body and provoke mal aire and the role of the shaman in solving illnesses of this nature. Like in our Asháninka community, also the Yanesha of Oxapampa have different kinds of expert healers where the 'tabaquero' belongs to a former stage of shamanism ('sheripiari', the Asháninka word for 'shaman', means 'tobacco drinker'), while the 'curanderos' which work with Ayahuasca and with other ways of divination are seen with diffidence [60].

## Conclusions

In the community of Bajo Quimiriki, despite the vicinity to the city of Pichanaki, traditional plant knowledge has still a great importance in the daily life: 402 medicinal plants were indicated by the informants for the treatment of 155 different ailments and diseases. Of these, 'mal aire', malaria, diarrhoea, 'chacho', headache and intestinal parasites were reported as the most common among community inhabitants.

The majority of medicinal plants indicated by the informants were herbs, prepared through decoction and administered externally, although shrubs, trees, lianas, epiphytes and arborescent ferns were also indicated. Other traditional ways of preparation included steam baths, always administered by women. The majority of the medicinal plants were found in the forest, followed by the homegardens and along the river. Among the medicinal plants cultivated in the homegardens, two particular categories of plants called *pinitsi *and *ivenki *(*Cyperus *spp.) had an important cultural value, often for magic-protective use in the household.

Exotic plants represented a minor component of the local pharmacopoeia, being limited to 5 species (2%). Their uses did not differ from the ones made of local species.

Medicinal plants knowledge was not restricted to the specialists, but included men, women and also children. There was a significant correlation between age of informants and knowledge of medicinal plants and during the forest walks women could identify more medicinal plants than men. However the differences in knowledge of medicinal plants were also related to personal interest and relation with a local healer. Children could list almost half of the most well-known medicinal plants in the community, but they knew them almost exclusively by their Spanish name. The specialists of the community played a special role dealing with illnesses caused by spirits that inhabited the forest and water or to diagnose the causes of diseases. The shaman and the midwife in the community received mainly non-indigenous customers, showing a progressive shift of their role towards a source of income. The medicinal plants used in this Asháninka community overlapped by 16% with the medicinal plants reported in three Yanesha communities in the Pasco Region of Peru. The two ethnic groups shared many believes and diseases. This is interesting considering that two of the Yanesha communities were located in remote areas. This fact and the fact that there was a minimum amount of exotic species in the local pharmacopoeia, suggests that despite the vicinity to a city, knowledge on medicinal plants use and traditional believes remain abundant. Moreover the medicinal plants are still available in the surrounding of the community. Documentation and quantification of their actual use could be subjects of future studies. A more accurate study focusing on medicinal plant knowledge in children with data on their family of origin could give a more clear idea of how knowledge is transmitted to the young generations and reveal if this process is threatened. It would be interesting to review the available literature on pharmaceutical properties of the medicinal plants that were most known in the community and of those that were used for similar purposes by the Yanesha. These plants might have a great potential for future drug development.

## Competing interests

The authors declare that they have no competing interests.

## Authors' contributions

GL carried out the fieldwork, analyzed the data and drafted the manuscript. MS and IT supervised the work at all its stages, contributing to its conception and design, interpretation of data and drafting of the manuscript. PM gave recommendations to the design of the fieldwork, revised critically the manuscript and provided writing assistance. All authors read and approved the final manuscript.

## Supplementary Material

Additional file 1 **Medicinal plants from the Native Community of Bajo Quimiriki**. The data provided represent the complete overview on the 402 collected medicinal plants: scientific names, collection numbers, vernacular names, habitat type, life form, use, plant parts used, preparation, route of administration and informants' number, gender and age. The data are provided in the form of a Microsoft Excel spreadsheet. Click here for file

Additional file 2 **List of Asháninka informants**. The list provides the names of the inhabitants of the Asháninka Native Community Bajo Quimiriki who participated in the various activities, sharing their knowledge on medicinal plants. Click here for file
