# Supplementary material for: Asháninka medicinal plants: a case study from the native community of Bajo Quimiriki, Junín, Peru
Source: J Ethnobiol Ethnomed. 2010 Aug 13;6:21. doi: 10.1186/1746-4269-6-21 (PMC2933607; doi:10.1186/1746-4269-6-21)
Supplement: Additional file 1 — Medicinal plants from the Native Community of Bajo Quimiriki. The data provided represent the complete overview on the 402 collected medicinal plants: scientific names, collection numbers, vernacular names, habitat type, life form, use, plant parts used, preparation, route of administration and informants' number, gender and age. The data are provided in the form of a Microsoft Excel spreadsheet. [file 1746-4269-6-21-S1.PDF]

Additional file 1. Medicinal plants from the native community of Bajo Quimiriki

Scientific name (Voucher N.)	Habitat type[a]	Status[b]	Asháninka name	Spanish name	Life form	Use	Plant part used	Preparation	Route of administration	No. of Inf.	Informants' gender and age
**DIVISION MAGNOLIOPHYTA**											
**Acanthaceae (6[c]/8[d])**											
Justicia appendiculata Vahl	F	w	Tsorishi		Herb	To loose weight	Leaves	Decoction	Oral	**5**	F: 71
(FL16, R36, WI02)						Relaxant	Leaves	Decoction	External		M: 27
			Hitinkishiretsi			Mal aire	Leaves	Decoction	External		F: 46<br>M: 32
								Steam bath	External		M: 19
			Toripenkishi			Fever		Decoction	External		M: 32
Justicia sp. 1 (FL13)	F	w			Herb	Mal aire	Leaves and stem	Steam bath	External	**1**	F: 71
Justicia sp. 2 (1102)	H	w	Chorishi		Herb	To strenghten elderly people	Leaves	Decoction	External	**1**	F: 30
Fittonia sp. (0513)	H	c	Kichonkashiri		Herb	General not well being	Leaves and stem	Decoction	External	**1**	F:23
Sp. 1	H	w	Pinitsi		Herb	To give birth rapidly	Leaves	Decoction	Oral	**4**	F: 23
(0510)						To bathe babies	Leaves	Decoction	External		F: 25
						Arcoiris	Leaves	Decoction	External		F: 25
						Mal aire	Leaves	Decoction	External		F: 25
						Scapular arthritis	Leaves	Decoction	External		M: 50
						Headache	Leaves	Decoction	Oral		M: 38
Sp. 2 (1103)	H	w	Atsarishiwenky		Herb	To give birth rapidly and not feel the pain	Leaves	Decoction	Oral		F: 30
**Amaranthaceae (1/4)**											
Iresine diffusa Humb. & Bonpl. ex Willd.	F	w	Popikishi		Herb	Stomach parasites	Leaves and stem	Decoction	Oral	**4**	F: 35, 55
(CA06, IS02, LI4, PE2)								Fresh	Oral		M: 55
			Tonkiroshi			Liver-complaints	Leaves and stem	Decoction	Oral		F: 36

			Kepishiri								
**Anacardiaceae (2/2)**											
Anacardium occidentale L. (2917)	H	c		Marañon	Tree	Uta (Leishmaniasis)	Fruit pericarp's oil	Fresh	External	**1**	M: 33
Tapirira guianensis Aubl. (R57)	F	w	Sanipan		Tree	To give birth without pain	Bark	Decoction	Oral	**5**	M: 27
						Fever	Leaves and bark	Steam bath	External		F: 36
						Headache	Leaves and bark	Steam bath	External		F: 36
						Nausea	Leaves and bark	Steam bath	External		F: 36
						To protect from Sorcery and illness	Leaves and bark	Steam bath	External		F:55
			Ontsamania			Wound healing	Leaves	Decoction	External		F: 71
							Bark	Fresh	External		F:35
						Uta (Leishmaniasis)	Bark	Fresh	External		F:35
**Annonaceae (1/3)**											
Annona muricata L. (0701,1803, 2906)	H	c		Guanabana	Tree	Stomach parasites	Leaves	Decoction	Oral	**6**	F: 21
						Gonorrhoea	Leaves	Decoction	External		M: 38, 27
									Oral		M: 27
						Ovary infection	Leaves	Decoction	Oral		F: 33
						Stomach ache	Leaves and bark	Decoction	Oral		M: 50
							Leaves	Decoction	Oral		M: 38
						Infection of urinary duct	Leaves	Decoction	External		F: 23
						Prostate	Leaves	Decoction	External		F: 23
**Apiaceae (1/1)**											
Eryngium foetidum L. (4023)	H	c		Sacha culantro	Herb	To give birth rapidly	Root	Decoction	Oral	1	F: 27
**Apocynaceae (5/7)**											
Asclepias curassavica L. (R29)	F	w	Shaoripini, Shaorishi, Kaporkishi	Leche malva	Herb	Wound healing	Latex	Fresh	External	**10**	M: 50, 27, 38, 69, 50 F: 55, 45, 24
			Ashi Avatharo	Mala leche		Uta (Leishmaniasis)	Latex	Fresh	External		F: 71
						Eye inflammation	Latex	Fresh	External		M: 19

Himatanthus sucuuba (Spruce ex Müll. Arg.) Woodson	F	w	Paréntsipa, Paiantsipa	Tree	Stomach parasites	Latex	Fresh	Oral	**5**	F: 45, 55
(EL4, IS3, PE5)			Kachónpitoki		Stomach ache	Twigs	Decoction	Oral		M: 50
					Mal aire	Leaves	Decoction	External		M: 69, 50
					Sorcery	Leaves	Decoction	External		M: 50
Tabernaemontana sp. (IS06)	F	w	Kachonpitoki	Tree	Diarrhoea	Stem	Decoction	Oral		F: 55
Sp.1 (3007)	H	w	Oje pari	Herb	Arcoiris	Root	Decoction	External	**1**	M: 50
Sp. 2 (R78)	F	w	Tsikontitsa	Herb	Headache	Latex	Fresh	Eye	4	M: 27<br>F: 55
					Snake bites	Leaves	Emplast	External		F: 36
			Pusanga		To attract women	Leaves	Decoction	External		F: 71
**Araceae (17/29)**										
Anthurium dombeyanum Brongn. ex Engl.	F	w		Herb	Snake bites	Aerial Roots	Decoction	Oral	**10**	M: 27
(R23, R64)						Leaves	Emplast	External		M: 28
			Maranketsa			Leaves and aerial roots	Emplast	External		M: 50
					Mal aire	Leaves	Decoction	External		F: 71
					To attract men	Leaves	Steam bath	External		F: 71
			Isavanare		Headache	Leaves	Decoction	External		M: 50
						Leaves and root	Decoction	External		F: 55
							Steam bath	External		M: 50
			Porenkishi		To enhance hair growth	Leaves and stem	Decoction	External		M: 32
			Muetashipani		Stomach parasites	Stem	Decoction	Oral		M: 55
					Infection of urinary duct	Leaves and root	Decoction	Oral		F: 36
					Stomach ache	Stem	Decoction	Oral		M: 27
			Tampiashi		Cough	Leaves	Decoction	External		F: 71
					Seeing shadows	Leaves	Decoction	External		F: 71
Anthurium kunthii Poepp. (R22)	F	w	Patakaroshi	Epiphyte	Stomach parasites	Stem	Decoction	Oral	**4**	M: 27

			Vairontsi	Panca panca poderosa		To enhance women fertility	Fruit	Fresh	Oral		M: 32
						Stomach ache	Stem	Fresh	Oral		F: 55
						To abort	Stem	Fresh	Oral		F: 71
Anthurium macleani Schott	F	w	Pitokashniro	Trompa de mujer	Herb	Menstruation pain	Leaves	Decoction	Oral	**4**	M: 27
(R66)						Ovary inflammation	Leaves	Decoction	Oral		M:27<br>F:36
			Ashiomihe amuito			Stomach acidity	Stem	Infusion	Oral		F: 55
			Irasancane Pawá			To get ones spirit back	Leaves and stem	Decoction	External		F: 71
Anthurium pentaphyllum G. Don<br>(2815)	F	w		Orejita de burro	Epiphyte	Pains in the body	Leaves and stem	Decoction	External	**1**	F:30
Anthurium polydactylum Madison<br>(PE24)	F	w	Choritishi		Herb	Cramps	Leaves and stem	Decoction	External	**1**	M:55
Dieffenbachia costata Klotzsch ex Schott	F, H	w, c	Hentsiri	Ajo macho	Herb	Bronchitis	Stem	Decoction	Oral	**23**	M: 38<br>F: 46, 42, 25
(0704, 0605, 2813, R38, R77)						To protect from sorcery and illness	Leaves	Fresh	External		M: 38, 27<br>F: 36
						Insect bites	Stem	Fresh	External		M: 27, 19, 38, 29, 27<br>F: 36, 23
						Snake bites	Stem	Fresh	External		M: 29, 27<br>F: 36, 30
						Cough	Stem	Decoction	Oral		M: 55, 69<br>F:55, 71, 35, 28
						Osteoarthritis	Stem	Decoction	External		F: 55
						Wound healing	Stem	Fresh	External		F: 35
						Influenza	Stem	Steam bath	External		M: 32, 29<br>F: 46
						Toothache	Stem	Fresh	External		F: 38, 25

Homalomena crinipes Engler	F	w	Kainequiroqui	Pituca de monte	Herb	To clean the stomach	Tuberous rhizome	Fresh	Oral	**15**	M:27
(R4, R50)						Toothache	Tuberous rhizome	Fresh	External		M:50
			Manihishi			To abort	Tuberous rhizome	Decoction	Oral		M: 27<br>F:36, 71, 35, 30, 42
			Pitokaniro<br>Kainto<br>Tanaroshi			To protect teeth	Stem	Heated	External		M: 55<br>F: 46, 55, 35, 38, 25
			Pitokashi			Stomach parasites	Tuberous rhizome	Fresh	Oral		M:57
								Infusion	Oral		M: 69<br>F: 46
Monstera adansonii Schott	F	w	Azonki		Epiphyte	Ulcers	Stem	Decoction	Oral	**4**	M: 32
(R21)				Panca panca del monte		To enhance beard growth	Stem	Emplast	External		M: 32
						Relaxant	Stem	Steam bath	External		F: 36
				Costilla de Adán		Osteoarthritis	Stem	Steam bath	External		F: 36
						To abort	Stem	Fresh	Oral		F: 71
				Pituca selvaje		To disinflamate cysts	Fruit	Fresh	Oral		M: 27
Philodendron brandtianum K.Krause	F	w	Sampetacash		Herb	Browses and swellings	Leaves and stem	Decoction	External	**5**	M: 27<br>F:36
(R71, R62)			Kaintotza			Internal inflammations	Stem	Decoction	Oral		M: 27
			Kainto			Internal pain 'vaso'	Leaves and stem	Decoction	Oral		F: 55
			Meritsonsokishi			Pains in the body	Leaves and stem	Decoction	External		F: 55
						Stomach parasites	Petiole	Cold water extract	Oral		F: 71
			Pitokanashi			Stomach ache	Stem	Cold water extract	Oral		M: 32
						Heart-complaints	Stem	Cold water extract	Oral		M: 32
Philodendron deflexum Poepp. ex Schott	F	w		Pituca selvaje, Pituca del monte	Herb	Stomach parasites	Stem	Fresh	Oral	**4**	M: 27

(R6, PE31)			Sampetacashi			Browses and swellings	Leaves	Emplast	External		F: 36
			Kainto			Cough	Stem	Cold water extract	Oral		F: 55
						Cramps	Leaves	Fresh	External		M: 55
							Stem	Fresh	Oral		F: 71
			Tsairontsi			To enhance hair growth	Root	Decoction	External		M: 55
Philodendron ernestii Engl.	F	w	Kainto		Herb	Stomach parasites	Petiole	Cold water extract	Oral	**1**	F: 71
(FL25)											
Philodendron hylaeae G.S. Bunting	F	w			Epiphyte	Stomach parasites	Stem	Decoction	Oral	**1**	M: 19
(ROM7)											
Philodendron oligospermum Engl.	F	w			Herb	Internal pain 'vaso'	Leaves	Decoction	External	**1**	F: 36
(LI20)											
Philodendron plowmanii Croat	F	w		Pituca del monte	Herb	Anaemia	Stem	Decoction	External	**5**	M: 27
(R81)			Pitokashi			Stomach parasites	Stem	Fresh	Oral		M: 57
			Shiretsishi			Mal aire	Leaves and stem	Decoction	External		F: 55, 46
			Kainto			Seeing shadows	Leaves	Fresh	External		F: 71
Rhodospatha latifolia Poepp. & Endl.	F	w	Kainto Irashi Ashitane		Epiphyte	Stomach parasites	Stem	Fresh	Oral	4	M:27
(R82, R68)			Kaintotsa Kainto					Decoction	Oral		F: 55, 71
			Imokishi	Panca panca		Scapular arthritis	Stem	Fresh	External		M: 19
			Shi charishi			Fungal infections	Leaves	Decoction	External		M: 27
Syngonium podophyllum Schott	F	w	Kuritishi, Merithadeki		Herb	To correct irregular menstruation	Stem	Decoction	Oral	**12**	M: 27
(R9, 2814, DH09, DH04)							Leaves	Decoction	Oral		F: 55

			Meritsareki, Merishi			To extract caried teeth	Latex	Fresh	External		M: 50<br>F: 36
						Toothache	Latex	Fresh	External		F: 71, 30, 28
						Contraception	Leaves	Decoction	Oral		F: 45
			Kainto			Stomach parasites	Stem and root	Fresh	Oral		M: 69
							Stem	Fresh	Oral		M: 50
						Joint dislocations	Stem	Cold water extract	Oral		M: 26
			Kaintota			Anaemia	Leaves	Decoction	External		M:57
			Kaintoshi			Osteoarthritis	Leaves and stem	Emplast	External		M:57
						Browses and swellings	Leaves and stem	Emplast	External		M:57
	Xanthosoma poeppigii Schott	F	w	Kenashi	Panca panca	Epiphyte	Wound healing	Tuber	Emplast	External	**13**	M: 27, 29<br>F: 55, 45
	(R89)						Latex	Fresh	External		M: 55, 19, 38, 50, 32<br>F: 71, 45, 46
							Stem	Fresh	External		F: 35
						Bone fractures	Tuber	Emplast	External		F: 36
**Arecaceae (6/7)**											
	Astrocaryum sp.	F	w	Shishirishi		Shrub	Head lies	Leaves	Decoction	External	**4**	M: 55
	(PE37, ROM6)			Ashanke			Spider bites	Stem	Fresh	External		M:19
					Cola de caballo		To enhance hair growth	Leaves and stem	Decoction	External		M: 50
				Kío			Sorcery	Root	Decoction	External		F: 25
									Steam bath	External		F: 25
	Chamaedorea fragrans Mart. (AQ03)	F	w	Shia-shia		Tree	Warts	Flower	Fresh	External	**1**	F: 46
	Bactris gasipaes Kunth (DH05)	F	w	Kíri	Pijuayo	Tree	Stomach parasites	Root	Decoction	Oral	**1**	M: 50
	Geonoma sp. (PE26)	F	w	Chentipa		Shrub	To enhance women fertility	Root	Fresh	Oral	**1**	M: 55

Socratea exorrhiza (Mart.) H. Wendl. (WI17)	F	w	Chendero	Camonilla	Tree	Penis extender	Bark	Decoction	Oral	**1**	M: 29
Iriartea cf. *deltoidea*	F	w	Camona		Tree	To improve male libido	Root	Infusion	Oral	**3**	F: 71
(FL22)			Kamonashi			Skin spots	Bark	Decoction	External		M: 50
						Mal aire	Bark	Decoction	External		M: 50
						Fungal infections	Bark	Decoction	External		M: 50
						Penis extender	Root	Emplast	External		M: 69
**Asphodelaceae (1/1)**											
Aloe vera (L.) Burm. f.	H	c		Sabila	Herb	To prevent hair loss	Leaves	Cold water extract	External	**1**	F: 46
(0611)						Skin spots	Leaves	Fresh	External		F: 46
**Asteraceae (31/46)**											
Acmella oleracea (L.) R.K.Jansen	F, H	w	Kobirikishi		Herb	Sunburn	Leaves	Emplast	External	**15**	M: 29
(WA4, 0203, PL22)								Decoction	External		M: 29
			Koveriki	Botón de oro		Pokio	Leaves	Decoction	Oral		M: 57, 50
							Leaves and stem	Decoction	External		F: 28
									External		M: 57, 50
						Browses and swellings	Leaves	Decoction	Oral		M: 57
									External		M: 57
								Emplast	External		M: 38
			Iovirikishte			Chacho	Leaves	Decoction	External		F: 25, 23
						Diarrhoea	Flowers	Fresh	External		F: 23
				Botoncillo		Insect bites	Flowers	Fresh	External		M: 27<br>F: 42
							Leaves	Heated	External		F: 25, 35, 36
						Toothache	Flowers	Fresh	External		M: 26
						Earache	Flowers	Fresh	External		M:40
			Kovirikishi			Arcoiris	Leaves	Heated	External		F: 25, 35, 36
Acmella sp. (WA04)	F	w	Kobirikishi		Herb	Sunburn	Leaves	Boiled emplast	External		M: 29

Ageratum conyzoides L.<br>(PA1)	F	w	Santapetashi		Herb	Stomach parasites	Leaves	Decoction	Oral	**1**	M: 38
Bidens pilosa L.	H, F, B	w	Tsideroqui		Herb	Burned skin	Leaves	Fresh	External	**10**	M: 27, 19<br>F: 24
(0207, R3, LI25, PL13, ROM1, FL3)								Decoction	External		F: 36, 35, 24
						To prevent formation of scars	Leaves	Fresh	External		M: 27
			Pichana			To give birth rapidly	Leaves and stem	Decoction	Oral		M: 50
			Pichanashi			To prevent hair loss	Stem	Decoction	External		M: 29
			Machancará				Leaves and stem	Decoction	External		M: 38<br>F: 46
			Pichana				Leaves, stem and root	Emplast	External		F: 36
			Machancará					Decoction	External		F: 55
			Machancará			Dandruff	Leaves and stem	Infusion	External		F: 46
			Machancará			Contraception	Leaves	Infusion	Oral		M: 50
			Machancará			To make babies walk fast	Root	Decoction	External		F: 71
						Skin spots	Leaves and stem	Decoction	External		F: 36, 35, 24
Bidens sp.<br>(0911)	H	w	Shilco	Manzanilla de monte	Herb	Skin rashes due to allergy	Leaves and stem	Decoction	External		F: 25
Bidens sp.<br>(FL03)	F	w	Chinchis		Herb	Fever	Leaves	Decoction	Oral	**1**	F: 71
Chaptalia nutans (L.) Pol.	F, H	w	Kepishiri	Diente de león	Herb	Malaria	Leaves and stem	Fresh	Oral	**11**	F: 30<br>M: 33, 55
(2811, 2908, R34)			Ketarishi	Lengua de perro		Stomach ache	Leaves	Fresh	Oral		M: 27<br>F: 55, 35
								Decoction	Oral		M: 29
				Chicoria		Diarrhoea	Leaves	Fresh	Oral		F: 36, 46<br>M: 55
						Stomach parasites	Leaves	Fresh	Oral		F: 36
							Root	Decoction	Oral		F: 45<br>M: 19

Clibadium sylvestre (Aubl.) Baill.	H	c	Huaco		Herb	Malaria	Leaves	Decoction	Oral	**5**	F: 46, 42, 25<br>M: 27
(0608, 0902)			Wacoshi			Wound healing	Leaves	Decoction	External		M: 50
Conyza sp.<br>(3010)	H	w	Sancashi		Herb	Stomach acidity	Leaves	Infusion	Oral	**1**	M: 50
Dichorisandra sp. (FL19)	F	w			Herb	To enhance women fertility	Root	Cold water extract	Oral	**1**	F: 71
Eclipta prostrata (L.) L.<br>(0908)	H	w		Botoncillo	Herb	Chacho	Leaves and stem	Decoction	External	**1**	F: 25
Hebeclinum macrophyllum (L.) DC.	F	w	Shawetashi	Amargón	Herb	Malaria	Leaves	Fresh	Oral	**14**	M: 27, 27
(R33, 0107, R7)			Kipishisati	Oja buena		Stomach ache	Leaves	Fresh	Oral		M: 27, 26, 50, 27
								Decoction	Oral		M: 69
						Infection of urinary duct	Root	Decoction	Oral		F: 71
						Liver-complaints	Leaves	Fresh	Oral		F: 36
						Stomach parasites	Leaves	Fresh	Oral		M: 55
						Chickenpox	Leaves	Decoction	External		F: 30
						Diarrhoea	Leaves	Fresh	Oral		F: 23, 25<br>M:27
						Stomach acidity	Leaves	Fresh	Oral		F: 23
						Colics	Leaves	Fresh	Oral		F: 25
Erechtites hieraciifolius (L.) Raf. ex DC. (0211, PL23)	H	w	Sancashi		Herb	Skin spots	Leaves	Fresh	External	**14**	M: 57, 26, 27, 38, 50, 27, 69<br>F: 30, 23, 25, 28
			Araincashi			Acne	Leaves	Emplast	External		M: 26
			Capencashi			Uta (Leishmaniasis)	Leaves	Heated	External		F: 42
			Shawetapetashi			To prevent hair loss	Leaves and stem	Decoction	External		F: 38
Mikania micrantha Kunth	B	w	Nihashi		Vine	Headache	Leaves and stem	Decoction	External	**10**	F: 35, 36, 25
(PL18)						Osteoarthritis	Leaves and stem	Decoction	External		F: 35, 36, 25
						Scapular arthritis	Leaves and stem	Decoction	External		F: 35, 36, 25

			Camotillo			Chacho	Leaves and stem	Decoction	External		F: 35, 36, 25<br>M: 69
			Korithmantashi			Sorcery	Leaves and stem	Decoction	External		F: 30
				Camotillo del monte		Arcoiris	Leaves and stem	Decoction	External		M: 27<br>F: 25, 42
			Oje torishi								
						Pokio	Leaves and stem	Decoction	External		M: 50<br>F: 42, 38
			Koritishi								
						Alcoholism	Leaves	Decoction	External		F: 23
						Acne	Leaves	Decoction	External		F: 38
			Koritsimantashi								
Mikania sp.<br>(R31)	F	w	Genitsa		Vine	Lack of appetite	Stem	Fresh	Oral	**1**	M: 27
Munnozia hastifolia (Poepp.) H.Rob. & Brettell<br>(R27)	F	w		Ala de murcielago	Herb	Cholera	Leaves	Fresh	Oral	**12**	F. 27
						Sorcery	Leaves and stem	Decoction	External		F: 55
						Kidney-complaints	Leaves	Fresh	Oral		F: 71<br>M: 57
						Gastritis	Leaves	Decoction	Oral		M: 19
						Wounds	Sap	Fresh	External		M: 38
							Leaves	Decoction	Oral		M: 32
				Alucema macho		Malaria	Leaves	Infusion	Oral		F: 25
						To stop dreaming dead people	Leaves	Decoction	Oral		F: 23
						Stomach ache	Leaves	Decoction	Oral		M: 50
						Mal aire	Leaves	Decoction	External		M: 50
						Fever	Leaves	Decoction	Oral		M: 38
						Cholesterol	Leaves	Decoction	Oral		M: 69
Porophyllum ruderale (Jacq.) Cass.	B	w	Samerentsishi		Herb	Stomach acidity	Leaves and stem	Decoction	Oral	**8**	F: 36, 35, 24, 42<br>M: 27
(PL12)			Samenposhi			To disinfect wounds	Leaves	Fresh	External		M: 50
			Kataroshi			Eye inflammation	Stem	Fresh	External		F: 30
			Kapencashi			Skin spots	Leaves	Fresh	External		M: 69
Senecio sp.<br>(0101, PL20)	H, B	w		Arnica	Herb	Browses and swellings	Leaves and stem	Fresh	Oral	**4**	F: 71
								Emplast	External		F: 71, 25

						Chacho	Leaves	Decoction	External		M: 69
				Llantén		Stomach ache	Leaves and stem	Decoction	Oral		M: 50
Spilanthes sp. (3011)	H	w	Kuviriki		Herb	Insect bites	Flower	Fresh	Oral	**1**	M: 50
Tagetes erecta L. (0202)	H	w			Herb	Mal aire	Leaves and Stem	Decoction	External	**1**	M: 57
Tessaria integrifolia Ruiz & Pav.	H, B	w	Shimagorishi	Pajaro bobo	Herb	Kidney-complaints	Leaves	Decoction	Oral	**10**	M: 27<br>F: 71
(0102, PL21)								Fresh	Oral		M: 69
						Ovary inflammation	Leaves and stem	Decoction	Oral		F: 36, 35, 24
								Decoction	External		F: 36, 35, 24
						General not well being	Stem	Steam bath	External		M: 50
								Decoction	External		M: 50
						Swollen feet	Leaves	Decoction	External		F: 30
						Pokio	Leaves	Decoction	External		F: 30
							Whole plant	Decoction	External		F: 28
						Chacho	Leaves	Decoction	External		F: 24
Trixis divaricata (Kunth) Spreng.	F	w	Nihashi		Herb	Arcoiris	Leaves	Decoction	External	**2**	M: 27
(R16)						Mal aire	Leaves	Steam bath	External		F: 36
Vernonia sp. (2801)	H	w	Poroquish		Shrub	Wound healing	Leaves	Fresh	External	**1**	M: 38
Vernonanthura patens (Kunth)H. Rob. (2809)	H	c	Pijoro		Tree	To bathe babies	Leaves	Decoction	External	**1**	F: 32
Sp. 1 (CA4)	F	w	Sancashi		Herb	Skin spots	Leaves	Fresh	External	**1**	F: 35
Sp. 2	F	w	Lechugia		Herb	Earache	Leaves and stem	Steam bath	External	**6**	M: 57
(DH1)						Cough	Leaves	Fresh	Oral		M: 69
						Osteoarthritis	Leaves	Fresh	External		M: 50

				Amargón		Diarrhoea	Leaves	Decoction	Oral		F: 23
						To prevent hair loss	Leaves and root	Decoction	External		F: 25
						Skin spots	Leaves	Fresh	External		F: 38
Sp. 3 (FL2)	F	w			Herb	Liver-complaints	Leaves	Fresh	Oral	**1**	F: 71
						Earache	Leaves	Steam bath	External		F:71
Sp .4 (PE1)	F	w	Pijoro	Tucunai	Tree	Eye inflammation	Stem	Fresh	Eye	**3**	M:55
						Liver-complaints	Root	Decoction	Oral		M: 55
						Emesis	Leaves	Decoction	Oral		M: 50
						Headache	Leaves	Decoction	External		M: 50
				Matico del monte		Mal aire	Leaves	Decoction	External		M: 69
Sp. 5 (R43)	F	w	Pijoro	Tucunai	Tree	Headache	Stem	Fresh	Eye	**1**	M: 27
Sp. 6 (DH2)	F	w	Pokekishi		Herb	Colics	Leaves	Fresh	Oral	**1**	M: 57
Sp. 7 (0210)	H	w	Pakoiashi		Herb	Fever	Leaves	Decoction	External	**1**	M: 57
						Influenza	Leaves	Decoction	External		M: 57
**Bignoniaceae (5/5)**											
Jacaranda copaia D.Don (R93)	F	w	Tsirapa		Tree	Uta (Leishmaniasis)	Leaves and bark	Ashes	External	**3**	M: 27 F: 55
			Kontamania			Wound healing	Leaves and bark	Decoction	External		F: 71
Mansoa alliacea (Lam.) A.H. Gentry (4022)	H	c		Ajo sacha	Liana	Mal aire	Leaves and stem	Steam bath	External	**1**	F: 32
Tynanthus polyanthus(Bureau) Sandwith (R69)	F	w	Emopari		Liana	Stomach ache	Root	Fresh	Oral	**7**	M: 27
			Tsinitsipari			Babies wetting the bed	Root	Fresh	Oral		F: 36
						Stomach parasites	Root	Fresh	Oral		M: 55
			Shirotoshi	Sigueme sigueme		To attract women	Leaves	Fresh	External		M: 57

						Prostate	Stem	Decoction	Oral		F: 55
			Pusanga			To bring good luck	Leaves	Fresh	External		F: 71
Sp. 1<br>(PE10)	F	w	Parivanashi		Herb	To make teeth fall	Leaves	Fresh	Oral	**1**	M: 55
Sp. 2<br>(R74)	F	w	Santimantico<br>Shimpanatsa		Herb	Influenza	Stem	Decoction	Oral	**7**	M: 27
							Leaves	Decoction	External		F: 36, 55<br>M: 55
								Steam bath	External		F: 45
						Malaria	Leaves and stem	Decoction	Oral		M: 27
			Pusanga			To attract the other sex	Leaves	Alcohol extract	External		F: 71
**Bixaceae (1/2)**											
Bixa orellana L.<br>(2810, 2907)	H	c		Achiote	Small tree	To prevent hair loss	Leaves	Decoction	External	**11**	M: 38, 50
						Eye infection	Petiole sap	Cold water extract	Eye		M: 33, 27<br>F: 23, 24
			Potsoti			Prostate	Root	Decoction	Oral		M: 69
							Leaves	Infusion	Oral		F: 42
							Leaves and bark	Decoction	Oral		M: 50
						Diarrhoea	Leaves and bark	Decoction	Oral		M: 50
						Wounds	Seed	Fresh	External		M: 27
							Root	Fresh	External		F: 30
						Stomach inflammation	Leaves	Decoction	Oral		M: 27
						Cancer	Leaves	Decoction	External		M: 26
						Kidney-complaints	Leaves and root	Decoction	Oral		F: 28
						To bathe babies	Leaves	Decoction	External		F: 23, 24
**Campanulaceae (2/2)**											
Centropogon cornutus (l.) Druce<br>(WI19)	F	w	Mathomero		Herb	To bathe babies	Leaves and stem	Decoction	Oral	**1**	M: 32
Centropogon sp. (WI15)	F	w	Tunkiriwanteshi	Picaflor del monte	Herb	Headache	Leaves	Fresh	Eye	**1**	M: 32
**Cecropiaceae (2/2)**											
Cecropia sp. 1	F	w		Yungul rojo	Tree	Liver-complaints	Leaves	Decoction	Oral	**1**	F: 35

(CA08)												
Cecropia sp. 2	(R13)	F	w		Yungul blanco	Tree	Memory problems	Bark	Decoction	Oral	**5**	M: 27
							Kidney-complaints	Leaf buds	Decoction	Oral		F: 36
								Root	Decoction	Oral		F: 71
							Prostate	Leaf buds	Decoction	Oral		M: 55
							Ulcers	Leaf buds	Decoction	Oral		M: 55
							Stomach tumor	Bark	Decoction	Oral		F: 55
**Chenopodiaceae (1/4)**												
Chenopodium ambrosioides L.		B	w		Paico	Herb	Stomach parasites	Leaves and stem	Fresh	Oral	**14**	F: 36, 35, 24, 46
(PL1, 0206, 0602, 0907)									Decoction	Oral		M: 69, 27<br>F: 23
							Colics	Leaves and stem	Decoction	Oral		M: 50, 57, 27<br>F: 36, 35, 24, 30, 28, 23
									Fresh	Oral		M: 27
							Fever	Leaves	Decoction	External		M: 38
							Diarrhoea	Leaves	Decoction	Oral		M: 38, 27
												F: 38
							To bathe babies	Leaves and stem	Decoction	External		F: 42
**Clusiaceae (3/3)**												
Vismia sp.1		F	w	Taronspini		Shrub	Burned skin	Leaves	Fresh	External	**1**	F: 46
(AQ14)												
Vismia sp.2	(PE30)	F	w	Kinchakishi		Shrub	Measles	Bark	Cold water extract	External	**1**	M: 55
							Chickenpox	Bark	Cold water extract	External		M: 55
Sp. 1		F	w			Shrub	Acne	Leaves	Decoction	External	**1**	M: 55
(PE33)									Heated	External		M: 55
**Combretaceae (1/1)**												
Terminalia sp.		F	w			Tree	Emesis	Leaves	Decoction	Oral	**2**	M: 27

(R86)							Mal aire	Leaves	Steam bath	External		F: 71
							Pains in the body	Leaves	Steam bath	External		F: 71
**Commelinaceae (7/7)**												
Commelina sp. 1	(0113)	H	c		Suelda suelda legitimo	Herb	Joint dislocations	Leaves and stem	Emplast	External	**1**	F: 71
Commelina sp. 2		H	c	Asonke	Suelda suelda	Herb	Bone fractures	Leaves and stem	Emplast	External	**1**	F: 30
(2102)							Gonorrhoea	Root	Decoction	External		F: 30
Commelina sp. 3	(PL3)	B	w	Vaviroshi	Tres corazones	Herb	Browses and swellings	Leaves and stem	Emplast	External	**5**	F: 36, 35, 25 M: 69
							Joint dislocations	Leaves and stem	Emplast	External		M: 26
Commelina sp.4		H	c			Herb	Menstruation pain	Leaves	Decoction	Oral	**1**	F: 21
(0705)												
Commelina sp. 5		F	w	Vavirotha		Herb	Browses and swellings	Leaves and stem	Emplast	External	**1**	M: 32
(WI3)							Bone fractures	Leaves and stem	Emplast	External		M: 32
Dichorisandra ulei J.F. Macbr.		H	c	Kasonki		Herb	Browses and swellings	Leaves and stem	Steam bath	External	**1**	F: 55
(4024)									Emplast	External		F: 55
Tradescantia sp.		F	w	Matincoritsa	Chupa sangre	Herb	Browses and swellings	Leaves and stem	Emplast	External	**6**	M: 27
(R2)				Kasonke	Suelda pichi				Decoction	Oral		M: 55
										External		M: 57, 32
							Bone fractures	Leaves and stem	Emplast	External		F: 36
									Decoction	Oral		F: 55
										External		M: 32
							To prevent ageing	Leaves and stem	Decoction	Oral		F: 36
**Convolvulaceae (1/1)**												
Ipomea sp.		H	w	Poinirokita		Herb	To correct irregular menstruation	Leaves	Decoction	Oral	**1**	M: 50
(3003)							Haemorrhage	Leaves	Decoction	Oral		M: 50
							Bone fractures	Leaves	Decoction	Oral		M: 50
							Tubercolosis	Leaves	Decoction	Oral		M: 50

**Costaceae (3/3)**											
	Costus sp. 1	H	w	Parianti	Platanillo	Herb	Mal aire	Leaves	Decoction	Oral	**1**	M: 50
	(R49)									External		M: 50
	Costus sp. 2	H	c	Asonke wenky		Herb	Kidney-complaints	Leaves and stem	Decoction	External	**1**	F: 33
	(2812)											
	Costus sp. 3	F	w			Herb	Mal aire	Leaves	Steam bath	External	**1**	F: 38
	(4016)											
**Cucurbitaceae (2/3)**											
	Cayaponia sp.	F	w	Shitorishi		Herb	Fungal infections	Leaves	Decoction	External	**1**	M: 32
	(WI11)											
	Momordica charantia Descourt.	H	c		Caiba blanca	Herb	Malaria	Leaves	Fresh	Oral	**13**	M: 69, 27, 38<br>F: 71, 42, 24, 33
	(0108, 2901)								Decoction	Oral		F: 28
					Caiba amarga		Stomach ache	Leaves	Fresh	Oral		M: 26<br>F: 71
									Decoction	Oral		M: 38
							Stomach acidity	Fruit	Fresh	Oral		M: 50
							Fever	Leaves	Decoction	Oral		M: 38
							Diarrhoea	Leaves	Decoction	Oral		M: 38
							Stomach parasites	Leaves	Fresh	Oral		F: 30
							Kidney-complaints	Fruit	Fresh	Oral		F: 38
							Mal aire	Leaves	Decoction	External		F: 23
**Cyclanthaceae (2/2)**											
	Cyclanthus bipartitus Poit.	F	w	Tsonaki		Shrub	To prevent hair loss	Leaves and stem	Decoction	External	**2**	M: 27
	(R87)						Sorcery	Leaves and stem	Steam bath	External		F: 71
							Mal aire	Leaves and stem	Steam bath	External		F: 71
	Carludovica palmata Griseb. (PE36)	F	w	Kepia	Bombonaje	Shrub	Skin spots	Fruit	Fresh	External	**9**	M: 55
							Mal aire	Leaves and stem	Decoction	External		M: 69
							Internal inflammations	Leaves and stem	Decoction	Oral		F: 28

						Babies wetting the bed	Leaves	Heated	External		F: 38
						Epilepsy	Stem	Fresh	Oral		F: 42
						Warts	Fruits	Emplast	External		F: 25, 30<br>M: 50, 26
**Cyperaceae (9/9)**											
Cymbopogon citratus (DC. ex Nees) Stapf	H	c		Hierba luisa	Herb	Stomach ache	Leaves	Infusion	Oral	**1**	F: 23
(0507)											
Cyperus sp. (PL10)	B	w	Amerishi		Herb	To abort	Leaves	Cold water extract	Oral	**7**	F: 36, 35, 25
			Ibenkiniro			Diarrhoea	Root	Decoction	Oral		F: 23, 30<br>M: 50
						Colics	Root	Decoction	Oral		M: 50
						To abort	Leaves	Decoction	Oral		M: 69
Eleocharis sp.1	B	w	Nihashi		Herb	Stomach ache	Leaves	Decoction	Oral	**5**	F: 35, 25, 36
(PL16)									External		F: 35, 25, 36
Eleocharis sp.2	B	w			Herb	Alcoholism	Leaves and stem	Decoction	Oral	**6**	F: 35, 25, 36
(PL19)				Cola de caballo		To enhance hair growth	Leaves and stem	Decoction	External		M: 50
						Liver-complaints	Leaves	Decoction	Oral		M: 27
			Ishpatonashi			Chacho	Leaves and stem	Decoction	External		F: 25
Rhynchospora sp.	F	w	Amerishi		Herb	To abort	Leaves	Decoction	Oral	**1**	M: 55
(PE12)											
Scleria secans Urb.	F	w	Amerishi	Cortadillo	Herb	To abort	Root	Decoction	Oral	**6**	M: 27, 29, 32<br>F: 36
(R18)							Leaves	Decoction	Oral		M: 55<br>F: 71
						Sorcery	Leaves	Steam bath	External		F: 71
Sp. 1	H	c	Chorana		Herb	Diarrhoea	Root	Decoction	Oral	**1**	M: 57
(0201)									External		M: 57
						Stomach ache	Root	Decoction	Oral		M: 57

Sp. 2 (2912)		H	c	Ivenki Piri-piri		Herb	To make babies sleep	Root	Decoction	External	**1**	F: 33
Sp. 3 (0209)		H	c	Ivenki Piri-piri		Herb	Fever	Root	Fresh	External	**8**	M: 57, 38
							Chacho	Root	Decoction	Oral		M: 69, 50 F: 25
							Pokio	Root	Decoction	External		M: 50
							Arcoiris	Root	Decoction	Oral		F: 25
							Headache	Root	Fresh	Eye		F: 42
							Emesis	Root	Fresh	Eye		F: 42
							Mal aire	Root	Decoction	External		M: 50, 38, 27 F: 30
							Sorcery	Root	Decoction	External		M: 50, 38
**Elaeocarpaceae (1/1)**												
Sloanea sp. (R53)		F	w	Chankeshi		Tree	Stomach parasites	Root and bark	Decoction	Oral	**1**	M: 50
**Euphorbiaceae (15/17)**												
Acalipha sp. 1 (ROM4)		F	w			Herb	Toothache	Latex	Fresh	External	**1**	M: 19
Acalipha sp.2	(R12)	F	w	Matiki Kichonkari		Herb	Haemorrhage	Leaves	Decoction	Oral	**4**	M: 27 F: 36, 55, 71
				Matiki						External		F: 55
Alchornea sp. (R72)		F	w			Tree	Stomach parasites	Latex	Fresh	Oral	**3**	M: 27
				Poroqui			Headache	Leaves	Decoction	Oral		F: 36
										External		F: 36
							Nausea	Leaves	Decoction	Oral		F: 36
										External		F: 36
				Kawana	Catagua		Sorcery	Leaves	Steam bath	External		F: 71
							Uta (Leishmaniasis)	Latex	Fresh	External		F: 71
Chamaesyce hirta (L.) Millsp. (4026)		H	w	Konarontsi		Herb	Wound healing	Latex	Fresh	External	**1**	F: 27

Croton lechleri Müll. Arg. (0609)	H	c	Irariki	Sangre de grado	Tree	Wound healing	Latex	Fresh	External	**12**	M: 69, 38, 27, 27<br>F: 46, 30, 42,<br>38, 28, 23, 24
						Ulcers	Latex	Fresh	Oral		M: 69<br>F: 27, 30
						Liver-complaints	Latex	Fresh	Oral		M: 50
						Uta (Leishmaniasis)	Latex	Fresh	External		F: 30
						Dysentery	Latex	Fresh	Oral		F: 38
						Disinflamation following parturition	Latex	Fresh	Oral		F: 24
Euphorbia heterophylla L.	F	w	Kataroshi		Herb	Wound healing	Latex	Fresh	External	**2**	M: 29, 33
(WA01, 2915)			Irashi Oje			Eye infection	Latex	Fresh	Eye		M: 29
			Ojé charishi								
Euphorbia sp. (0910, PL14)	H	w		Hierba con leche	Herb	Wound healing	Latex	Fresh	External	**1**	F: 25
Jatropha curcas L.	H	c		Piñon	Shrub	Diarrhoea	Leaves	Decoction	Oral	**9**	M: 33
(2919)						Uta (Leishmaniasis)	Latex	Fresh	External		M: 69<br>F: 28
						Stomach parasites	Seed	Fresh	Oral		M: 69, 50, 38<br>F: 30, 42, 23, 24
								Roasted	Oral		M: 27, 26
							Latex	Fresh	Oral		F: 28
						Liver-complaints	Seed	Fresh	Oral		M: 50
						Kidney-complaints	Seed	Fresh	Oral		M: 50
Manihot esculenta Crantz (2914)	H	c	Kaniri	Yuca	Shrub	To cure bites of dogs with rabies	Latex	Fresh	External	**1**	M: 33
Phyllanthus niruri L.	B	w		Chanca piedra hembra	Herb	Kidney-complaints	Leaves and stem	Decoction	Oral	**8**	F: 35, 36, 25, 28<br>M: 38, 69

(PL11)						Infection of urinary duct	Leaves and stem	Decoction	Oral		F: 35, 36, 25
						Pains in the body	Leaves and stem	Decoction	Oral		M: 50
						Browses and swellings	Stem	Emplast	External		M: 26
Phyllanthus orbiculatus Rich.	H	w	Chanca piedra		Herb	Kidney-complaints	Leaves and stem	Decoction	Oral	**1**	F: 38
(4010)											
Phyllanthus sp.	H	w	Tonpetzpari		Herb	To prevent hair loss	Root	Decoction	External	**1**	M: 33
(2921)											
Ricinus communis L.	B	w		Higuerilla	Shrub	Swollen feet	Leaves	Decoction	External	**6**	F: 36, 35, 24
(PL8)						Liver-complaints	Leaves and stem	Fresh	Oral		M: 69
			Morinaki			Mal aire	Leaves and stem	Decoction	External		M: 50<br>F: 28
			Jahiapa			Browses and swellings	Leaves and stem	Decoction	External		F: 24
Sp. 1	F	w	Chamairo		Liana	Insect bites	Bark	Fresh	External		M: 27
(R17)											
Sp. 2	F	w	Shawetapini	Amargón	Herb	Cholera	Leaves	Decoction	Oral	**5**	M: 27
(R88)						Malaria	Leaves	Decoction	Oral		M: 27, 55
			Sanipancash			Mal aire	Leaves	Decoction	Oral		F: 36
									External		F: 36
						Stomach parasites	Leaves	Fresh	Oral		M: 55, 19<br>F: 45
						Stomach acidity	Leaves	Decoction	Oral		F: 55
				Amargón blanco		Vaginal infection	Leaves	Decoction	Oral		F: 71
**Fabaceae (9/10)**											
Calliandra angustifolia Spruce ex Benth.	H	c	Quante	Bobinsana	Shrub	To strenghten newborn babies	Leaves, bark and root	Decoction	External	**2**	F: 36, 46
(1002, 0604)						To make babies walk fast	Leaves	Decoction	External		F: 46
						To strenghten elderly people	Leaves	Decoction	External		F: 46
Calliandra sp.	F	w	Kamantspini		Shrub	Influenza	Leaves	Decoction	External	**1**	M: 55
(PE8)											

Desmodium sp.1		F	w	Tsirerokishi		Herb	Kidney-complaints	Leaves and stem	Decoction	Oral	**1**	M: 29
(WA14)							Mal aire	Leaves	Decoction	External		M: 69
					Pinitsi del monte		Disinflamation following parturition	Leaves	Decoction	Oral		F: 28
					Shirco de bejuco		Haemorrhage	Leaves and stem	Decoction	Oral		F: 30
							Gastritis	Root	Fresh	Oral		M: 26
							Pains in the body	Leaves and stem	Decoction	Oral		M: 50
Desmodium sp.2	(WI06)	F	w	Itoshi Maranki	Cabezita de culebra	Herb	Snake bites	Leaves and stem	Decoction	External	**1**	M: 32
Desmodium sp.3	(4013)	F	w		Tsireroqui que gatea	Herb	Menstruation pain	Leaves	Decoction	Oral	**1**	F: 38
							Kidney-complaints	Leaves	Decoction	Oral		F: 38
Inga sp.		F	w	Intsipatsa		Tree	Haemorrhage	Bark	Decoction	Oral	**1**	M: 55
(PE25)								Root	Decoction	Oral		M: 55
Inga sp.	(R35)	F	w			Tree	Pain in the muscles after work	Bark	Decoction	Oral	**8**	M: 27, 69
				Tsiroqueshi			Uta (Leishmaniasis)	Bark	Heated	External		M: 55
									Fresh	External		F: 45
				Entsipashi	Pacai		Stomach parasites	Root	Infusion	Oral		F: 55
								Bark	Cold water extract	Oral		F: 71
				Paiarishi	Matico del monte		Mal aire	Leaves	Decoction	External		M: 50, 69
				Nihashi			To bathe babies	Leaves	Decoction	External		M: 26
							Chacho	Leaves	Decoction	External		M: 69
Phaseolus sp.		H	c		Frijol	Herb	Headache	Leaves	Fresh	Eye		F: 25
(0905)												
Sp. 1		F	w	Taankerontsi		Herb	Against laziness in children	Leaves	Decoction	External	**1**	M: 55
(PE9)												
**Gesneriaceae (4/4)**												
Alloplectus sp.		F	w	Ibini Maranki		Herb	Snake bites	Leaves	Boiled emplast	External	**1**	M: 27

(R26)											
Corytoplectus speciosus (Poepp.) Wiehler	F	w		Chalanca hembra	Herb	Mal aire	Leaves and stem	Steam bath	External	**5**	M: 27
(R76)							Leaves	Decoction	External		F: 36
						Dysentery	Leaves and stem	Decoction	Oral		M: 27
			Manitishi			To make babies walk fast	Leaves	Decoction	External		F: 55
				Chalanca roja		Seeing shadows	Leaves	Decoction	External		F: 71
							Stem	Decoction	Oral		F: 71
						Arcoiris		Boiled emplast	External		M: 38
						Pokio		Boiled emplast	External		M: 38
Diastema comiferum Benth. ex Walp.	F	w	Koshiripini		Herb	Spider bites	Leaves	Boiled emplast	External	**1**	F: 36
(4019)											
Gloxinia perennis Fritsch	F	w	Carpunto		Herb	To give birth rapidly	Leaves and stem	Decoction	Oral	**1**	F: 73
(4001)											
**Haemodoraceae (1/5)**											
Xiphidium caeruleum Aubl.	F, H	w	Chapapetashi	Cola de gallo	Herb	Bone fractures	Leaves	Emplast	External	**11**	M: 50
(R55,0606, R41, 4004, 4007)						Joint dislocations	Leaves	Emplast	External		M: 50, 27
						Browses and swellings	Leaves	Emplast	External		M: 50, 29<br>F: 55, 46
								Steam bath	External		F: 46
						To strenghten hair	Leaves	Decoction	External		M: 29
						To enhance hair growth	Leaves	Fresh	External		M: 32<br>F: 36, 71
						"Mal agua"	Leaves	Steam bath	External		M: 27
						To bathe babies	Leaves	Decoction	External		M: 27
**Iridaceae (1/2)**											
Eleutherine bulbosa (Mill.) Urb. (2103, 2909)	H	c		Cebollita	Herb	Contraception	Tuberous rhizome	Decoction	Oral	**2**	F: 30
						Haemorrhage	Tuberous rhizome	Fresh	External		M: 33
**Lamiaceae (2/2)**											

Ocimum basilicum L. (R46)	H	w		Albahaca	Herb	Influenza	Leaves	Steam bath	External	**1**	M: 27
Sp. 1 (0212)	H	w	Pinitsi		Herb	Headache	Leaves	Decoction	Oral	**8**	M: 57
						Chacho	Leaves	Decoction	Oral		M: 69, 38, 50
									External		M: 69
						To give birth rapidly	Leaves	Decoction	Oral		F: 30
						Mal aire	Leaves	Decoction	External		F: 30 M: 38, 50
			Pusanga			To attract women	Leaves	Alcohol extract	External		M: 26, 27
						Sorcery	Leaves	Decoction	Oral		M: 38, 50
									External		F: 42
			Piri-piri			Pokio	Leaves	Decoction	Oral		M: 50
**Lauraceae (1/1)**											
Aniba canelilla (Kunth) Mez (R65)	F	w	Metá	Arbol de canela	Tree	Stomach ache	Bark	Decoction	Oral	**6**	M: 27, 55, 29 F: 71, 35
						Diarrhoea	Bark	Decoction	Oral		F: 36
**Malpighiaceae (1/2)**											
Banisteriopsis caapi (Spruce ex Griseb.) C.V. Morton (2501, 2923)	H	c	Kamarampi	Ayahuasca	Liana	To connect with the spirits of the forest	Stem	Decoction	Oral	**6**	M: 33, 53
						To see other places	Stem	Decoction	Oral		M: 33, 53
						To diagnose illnesses	Stem	Decoction	Oral		M: 33, 53
						Cancer	Stem	Decoction	Oral		M: 69
						Arcoiris	Stem	Emplast	External		M: 50
						To see who is the responsible for a sorcery	Leaves	Decoction	Oral		M: 38
						Against general not well being	Leaves and bark	Decoction	External		F: 42
**Malvaceae (7/9)**											
Gossypium barbadense L. (0508, 0906)	H	c	Ampehi	Algodón	Small tree	To strenghten newborn babies	Leaves	Decoction	External	**2**	F: 25, 23
						Acne	Flower bud	Fresh	External		F: 25
Malachra alceifolia Jacq. (0702)	H	c		Malva	Herb	To clean the stomach	Leaves	Decoction	Oral	**1**	F: 21

Malachra ruderalis Gürke (0502)	H	c		Malva	Herb	Fever	Leaves	Decoction	External	**1**	F: 23
Sida rhombifolia L. (WA02, 2924)	F	w	Pichanashi		Herb	To prevent hair loss	Stem	Decoction	External	**2**	M: 29<br>F: 33
Sp.1 (0610)	H	c		Alucema	Herb	Colics	Leaves	Cold water extract	Oral	**1**	F: 45
Sp.2 (LI1)	F	w			Herb	Mal aire	Leaves	Decoction	External	**1**	F: 36
Sp. 3				Guinda blanca	Herb	Kidney-complaints	Leaves	Fresh	Oral	**1**	M: 55
(PE38)	F	w		Guindillo			Root	Fresh	Oral		M: 55
**Marantaceae (3/3)**											
Calathea sp. 1	F	w	Tsaviropa		Herb	Seeing shadows	Leaves	Decoction	Oral	**3**	M: 27
(R59)			Maniroshi	Platanillo					External		M: 27<br>F: 55, 71
Calathea sp. 2	F	w	Porenki		Herb	To strenghten newborn babies	Leaves	Decoction	External	**3**	M: 27
(R85)						Stomach inflammation	Stem	Fresh	Oral		F: 36
						Chacho	Root	Decoction	External		F:55
						Arcoiris	Root	Decoction	External		F:55
						Pokio	Root	Decoction	External		F:55
Sp. 1 (R40)	F	w	Porenki		Herb	Arcoiris	Leaves and root	Decoction	External	**1**	M: 27
**Melastomataceae (4/4)**											
Miconia sp. 1 (WA13)	F	w	Ahitsirishi		Shrub	Toothache	Leaves	Fresh	External	**1**	M: 29
Miconia sp. 2 (WI7)	F	w	Ampemanpoki		Shrub	Stomach parasites	Leaves	Decoction	Oral	**1**	M: 32
Miconia sp. 3	F	w	Shiretsishi		Shrub	Sorcery	Leaves	Decoction	External	**1**	F: 46

(EL6)											
Sp. 1<br>(LI16)	F	w			Shrub	To protect teeth	Leaves	Fresh	Oral	**1**	F: 36
**Meliaceae (1/1)**											
Sp. 1<br>(WI09)	F	w	Tsiwaki		Shrub	Mal aire	Leaves	Decoction	External	**1**	M: 32
**Menispermaceae (3/6)**											
Abuta grandifolia (Mart.) Sandwith<br>(WI14)	F	w	Sankiropana		Shrub	Influenza	Leaves	Decoction	External	**1**	M 32
Cissampelos tropaeolifolia DC.<br>(2802)	H	w	Matoshi		Herb	Acne	Leaves	Fresh	External	**1**	M: 38
Chondrodendron tomentosum Ruiz & Pav.	F, H	w	Nasankane tasorentsi	Corazón de Jesus	Vine	Stomach ache	Root	Fresh	Oral	**4**	M: 27
(R45, EL3, FL17, 2818)			Sarivana			Cholera	Root	Decoction	Oral		F: 45
						Stomach parasites	Root	Infusion	Oral		F: 71
			Kepishipari			Diarrhoea	Root	Fresh	Oral		F: 32
**Moraceae (3/3)**											
Artocarpus altilis (Parkinson) Fosberg<br>(2928)	H	c		Arbol del pan	Tree	Stomach parasites	Latex	Fresh	External	**1**	M: 33
Brosimum rubescens Taub.	F	w			Tree	Skin rashes	Latex	Fresh	External	**3**	M: 27
(R73)						Osteoarthritis	Leaves	Heated	External		F: 36
						To make babies walk fast	Leaves	Decoction	External		F: 35
Ficus sp.<br>(AQ05)	F	w	Ataparo		Shrub	Tumors	Leaves	Decoction	Oral	**1**	F: 46
**Musaceae (1/1)**											
Sp. 1	F	w	Kainto		Herb	Influenza	Leaves	Decoction	External	**3**	F: 46

(AQ10)						Stomach parasites	Leaves and stem	Fresh	Oral		M: 50, 69
**Myrtaceae (1/1)**											
Psidium guajava L.	H	c	Komashiki	Guajaba	Small tree	Diarrhoea	Leaves	Decoction	Oral	**1**	M: 33
(2930)											
**Nyctaginaceae (2/3)**											
Mirabilis jalapa L.	H	c	Kaniriaki		Shrub	To prevent white hair	Root	Decoction	External	**1**	F: 33
(2916, 4020)						To protect teeth	Root	Decoction	External		F: 33
Neea sp.	F	w			Shrub	Mal aire	Leaves	Steam bath	External	**1**	F: 36
(LI3)											
**Onagraceae (1/2)**											
Ludwigia peploides (Kunth) P.H. Raven	B	w	Nihashi	Bejuco de agua	Herb	Swollen feet	Leaves and stem	Decoction	External	**2**	F: 35, 36, 25
(PL04, PL17)			Iovirikishite			Chacho	Leaves	Decoction	External		M: 69<br>F: 25
						Pokio	Leaves	Decoction	External		F: 28
							Leaves and stem	Decoction	Oral		F: 30
								Boiled emplast	External		F: 42<br>M: 38
						Arcoiris	Leaves	Decoction	External		F: 28, 25
						Fungal infections	Leaves	Boiled emplast	External		F: 42
				Millonario		To live long	Leaves	Decoction	External		F: 23
			Toniroshi			Stomach ache	Leaves and stem	Infusion	Oral		M:50
**Orchidaceae (2)**											
Vanilla sp.	F	w	Kachonarishi		Vine	Stomach acidity	Leaves	Decoction	Oral	**1**	M: 29
(WA12)											
Sp. 1	F	w	Pusanga		Herb	To attract the other sex	Leaves and stem	Decoction	External	**1**	F: 71
(FL24)							Flowers	Fresh	External		F: 71
**Phytolaccaceae (3)**											
Petiveria alliacea L. (0104, 1804)	H	w		Mocura	Herb	To give birth rapidly and not feel the pain	Leaves	Decoction	Oral	**2**	F: 71

						Cramps	Root	Decoction	Oral		M: 38
						Stomach inflammation	Root	Decoction	Oral		M: 38
Petiveria sp.<br>(2101)	H	c	Shintsiwenky		Herb	To make babies sleep	Leaves	Fresh	Eye	**1**	F: 30
Phytolacca rivinoides Kunth & C. D. Bouché<br>(AQ12)	F	w			Herb	Skin spots	Fruit	Fresh	External	**1**	F: 46
**Piperaceae (11)**											
Lepianthes sp.<br>(LI5)	F	w	Sampetacashi		Herb	Browses and swellings	Leaves	Emplast	External	**1**	F: 36
Peperomia verschaffeltii Lem.<br>(R20, 4018)	F	w	Patakaroshi		Herb	Browses and swellings	Leaves	Emplast	External	**4**	F: 36, 35
						Haemorrhage	Leaves	Decoction	Oral		F: 55
			Kainto pirioki			Heart-complaints	Leaves	Fresh	External		F: 71
						For women to produce milk	Leaves	Decoction	Oral		F: 36
Peperomia sp. (R79)	F	w	Kachonirenchishi		Herb	Stomach acidity	Leaves	Fresh	Oral		M: 27<br>F: 55
			Kachonerishi			Pains in the body	Leaves	Fresh	External		F: 71
Piper aduncum L.<br>(R10,0603, 2806)	F, H	w,c	Huarantsishi	Matico	Small tree	Wound healing	Leaves	Decoction	External	**14**	M: 27
								Heat up the leaves and rub on the affected area	External		M: 27
								Steam bath	External		M: 27
						High Fever	Leaves	Decoction	Oral		M: 55
									External		F: 35, 45
						Ulcers	Leaves	Decoction	Oral		M: 50
						Mal aire	Leaves	Steam bath	External		M: 19<br>F: 36, 71
								Decoction	External		M: 38
								Decoction	Oral		M: 38<br>F: 55
						Vaginal infection	Leaves	Decoction	External		F: 35

						Sorcery	Leaves	Decoction	Oral		F: 46
						Menstruation pain	Leaves	Decoction	Oral		M: 32
						Cramps	Leaves	Decoction	Oral		M: 32
						Emesis	Leaves	Decoction	Oral		M: 19<br>F: 46
						Stomach ache	Leaves	Decoction	Oral		M: 19
							Twigs	Decoction	Oral		F: 36
						Cough	Leaves	Decoction	Oral		M: 38
						To disinfect wounds	Leaves	Decoction	External		M: 38
						Fungal infections	Leaves	Decoction	External		M: 38
Piper peltatum (L.) Miq.<br>(2911, 0114, 4003)	H	c	Tsibetashi	Matico hembra	Herb	To give birth without pain	Leaves	Decoction	Oral	**3**	M: 33
						To release the placenta after giving birth	Leaves	Decoction	Oral		F: 21, 30
Piper cf. *longestylosum*<br><br>(EL09)	F	w	Kamaritonkashi		Shrub	Mal aire	Leaves	Decoction	External	**1**	F: 45
Piper sp. 1<br>(R51)	F	w			Shrub	Mal aire	Leaves	Decoction	External	**1**	M: 50
Piper sp. 2<br>(DH7)	F	w	Shiritsitonkishi		Tree	Mal aire	Leaves	Decoction	External	**1**	M: 50
Piper sp. 3<br><br>(EL10)	F	w	Mashirotonpishi		Shrub	Mal aire	Leaves	Decoction	External	**1**	F: 45
Piper sp. 4<br>(R52)	F	w			Shrub	Sorcery	Leaves	Decoction	External	**1**	M: 50
Piper sp. 5<br>(LI14)	F	w			Shrub	Heart-complaints	Leaves	Decoction	External	**1**	F: 36
Piper sp. 6	H	w	Unkiro Wantikishi		Shrub	Headache	Leaves	Decoction	External	**1**	M: 50

(3006)						Nausea	Leaves	Decoction	External		M: 50
Piper sp. 7	H	c	Matico		Shrub	Mal aire	Leaves	Steam bath	External	**1**	F: 25
(3703)						Menstruation pain	Leaves	Decoction	Oral		F: 25
						Fever	Leaves	Decoction	External		F: 25
**Plantaginaceae (1)**											
Plantago major L.	H	w		Llantén	Herb	Disinflammatory	Leaves	Decoction	Oral	**8**	F: 21
(0904, 0707)									External		F: 24
						Liver-complaints	Leaves	Fresh	Oral		M: 69
								Decoction	Oral		M: 69, 27<br>F: 24
						Wounds	Leaves	Fresh	External		M: 69
								Heat up and emplast	External		M: 50
						Fever	Leaves	Infusion	Oral		F: 42
						Vaginal infection	Leaves	Decoction	External		F: 42
						Stomach ache	Leaves	Cold water extract	Oral		F: 28, 23
**Poaceae (8)**											
Acroceras cf. *zizanioides*	B	w	Porenki		Herb	Arcoiris	Root	Decoction	External	**5**	F: 25, 35, 36
(PL07)							Leaves	Decoction	Oral		F: 30
			Porenkishi			Headache	Leaves	Decoction	External		M: 50
									Oral		M: 50
Chusquea sp.	F	w	Suncari		Shrub	To make babies talk	Stem	Fresh	External	**3**	M: 27
(R63)						Headache	Leaves	Steam bath	External		F: 36
						Nausea	Leaves	Steam bath	External		F: 36
						Sorcery	Leaves	Decoction	External		F: 71
Eleusine indica Steud.	B	w	Kataroshi		Herb	To prevent hair loss	Stem	Emplast	External	**8**	F: 35, 36, 25
(PL15)								Decoction	External		M: 27, 50<br>F: 38
							Root	Decoction	External		M: 26
						Internal inflammations	Stem	Decoction	Oral		M: 38
						Eye inflammation	Sap	Fresh	Eye		F: 42

Paspalum conjugatum Bergius	H, F	w	Kataroshi		Herb	To prevent hair loss	Leaves	Decoction	External	**7**	M: 50<br>F: 71
(3002, FL27, R28, ROM2)						To prevent white hair	Leaves	Decoction	External		F: 42
						Eye infection	Sap	Fresh	External		M: 27, 19, 69, 26
						Pains in the body	Leaves and stem	Decoction	External		M: 50
Sp. 1	H	w	Nihawinki		Herb	Chacho	Stem	Decoction	External	**1**	F: 25
(0909)						Wounds	Stem	Decoction	External		F: 25
Sp. 2	H	c	Kishitsipini		Herb	To enhance hair growth	Stem	Infusion	External	**1**	F: 30
(1101)											
Sp. 3	F	w	Kío Tsirishi		Herb	To improve male libido	Stem	Decoction	Oral	**1**	F: 36
(LI23)											
Sp. 4	F	w	Tsivanashi	Piña del monte	Herb	Dandruff	Leaves	Decoction	External	**1**	M: 32
(WI5)						To prevent white hair	Leaves	Decoction	External		M: 32
**Polygalaceae (1)**											
Sp. 1	F	w	Tangarona		Tree	Cough	Bark	Decoction	Oral	**1**	M: 55
(PE34)											
**Polygonaceae (1)**											
Coccoloba sp.	F	w	Manehitsa		Liana	Ant bites	Stem	Fresh	External	**1**	M: 55
(PE15)											
**Rhamnaceae (1)**											
Ampelozizyphus sp.	F	w			Tree	Warts	Fruit	Fresh	External	**1**	F: 36
(LI15)											
**Rubiaceae (15)**											
Calycophyllum sp.	F	w	Sararo	Capirona de monte	Tree	To prevent white hair	Bark	Decoction	External	**8**	M: 27
(R58)						To boost immune system	Bark	Decoction	Oral		F: 36
						To become adult	Bark	Decoction	External		M. 29
						Fever	Bark	Decoction	External		F: 46

						Fatigue suppressor	Bark	Decoction	Oral		F: 55
						Wound healing	Bark	Decoction	External		F: 71
			Meshá			To prevent ageing	Bark	Decoction	External		F: 35<br>M: 55
Coccocypselum sp.<br>(AQ9)	F	w	Sharipini		Herb	Fungal infections	Leaves	Decoction	External	**1**	F: 46
Hamelia patens Jacq.<br>(2803, 2903)	H	w	Tsivanichetaki	Chupa Sangre	Shrub	To disinfect wounds	Leaves and stem	Heated	External	**11**	M: 38, 27<br>F: 28
						Browses and swellings	Leaves and stem	Heated	External		M: 33, 69
								Emplast	External		M: 38, 27, 26<br>F: 30, 42, 23
						Stomach ache	Leaves	Infusion	Oral		M: 50
						Headache	Leaves	Decoction	External		F: 38
Palicourea sp.<br>(EL7)	F	w			Shrub	Snake bites	Leaves	Roasted	External	**1**	F: 45
Psychotria poeppigiana Müll.Arg.<br>(PE19, AQ11)	F	w	Matapini		Shrub	Uta (Leishmaniasis)	Leaves	Heat up and squeeze in the wound	External	**4**	M: 50
			Ishipichankishi			Mal aire	Leaves and stem	Steam bath	External		F: 46
							Leaves	Decoction	External		M: 69
			Sharoshi			Headache	Leaves	Decoction	Oral		M: 50
									External		M: 50
Uncaria guianensis (Aubl.) J. F. Gmel.<br>(R19, PA3, R56, 4015, 4017)	F	w	Saventarotsa	Uña de gato	Liana	Osteoarthritis	Bark	Alcohol extract	Oral	**19**	M: 27, 29<br>F: 36
							Root	Alcohol extract	Oral		F: 55
			Tonpetsipari			Kidney-complaints	Bark	Alcohol extract	Oral		M: 27, 26<br>F: 36, 71
								Decoction	Oral		M: 69, 38<br>F: 40, 24
						Ulcers	Root	Decoction	Oral		M: 27, 55, 27
						Liver-complaints	Bark	Alcohol extract	Oral		M: 55, 50
							Leaves	Alcohol extract	Oral		F: 23

							Cold	Leaves	Steam bath	External		M: 27<br>F: 36
								Bark	Decoction	Oral		F: 40
								Bark	Alcohol extract	Oral		F: 24
							Cancer	Bark	Alcohol extract	Oral		F: 71
							Penis extender	Bark	Decoction	Oral		M: 38, 27
							Ovary inflammation	Bark	Decoction	Oral		F: 35
							Prostate	Bark	Alcohol extract	Oral		M: 50
									Decoction	Oral		F: 24
							To abort	Bark	Decoction	Oral		M: 27
							Stomach ache	Water contained in the stem	Fresh	Oral		F: 42
								Leaves	Alcohol extract	Oral		F: 23
							Inflammation	Water contained in the stem	Fresh	Oral		F: 28
Uncaria tomentosa (Willd.) DC.		F	w		Uña de gato	Liana	Arthritis	Bark	Decoction	Oral	**6**	F: 71
(PA4)					Uña de gato morada		Osteoarthritis	Bark	Alcohol extract	Oral		F: 71
							Kidney-complaints	Bark and leaves	Decoction	Oral		M: 69
								Bark	Decoction	Oral		M: 38<br>F: 24
									Alcohol extract	Oral		F: 71
							Stomach ache	Leaves	Alcohol extract	Oral		F: 23
							Inflammation	Bark	Decoction	Oral		M: 38
								Water contained in the stem	Fresh	Oral		F: 28
							Cold	Bark	Alcohol extract	Oral		F: 24
							Prostate	Bark	Decoction	Oral		F: 24
Sp. 1		H	w		Pinitsi de agua	Herb	Pokio	Leaves	Steam bath	External	**1**	M: 57
(0204)												
Sp. 2		F	w			Shrub	Headache	Leaves	Fresh	Eye	**1**	F: 45
(EL8)												
Sp. 3	(FL20)	F	w	Machancarai legitimo		Herb	To strenghten newborn babies	Leaves	Decoction	External	**1**	F: 71
							To make babies walk fast	Leaves	Decoction	External		F: 71

	Sp. 4 (FL21)	F	w			Herb	To loose weight	Leaves and stem	Decoction	Oral	**1**	F: 71
	Sp. 5 (IS4)	F	w	Shintsquirish		Herb	To make babies walk fast	Leaves	Decoction	External	**1**	F: 55
	Sp. 6 (IS8)	F	w	Marankeshi		Herb	Snake bites	Leaves	Emplast	External	**1**	F: 55
	Sp. 7 (PE16)	F	w	Tsonkiri		Shrub	Hernia	Bark	Emplast	External	**1**	M: 55
	Sp. 8	F	w		Cascarilla	Tree	Prostate	Bark and root	Alcohol extract	Oral		M: 27
	(R61)						Kidney-complaints	Bark and root	Alcohol extract	Oral		M: 27
								Bark	Decoction	Oral		F: 71
							Infection of urinary duct	Leaves and bark	Decoction	Oral		F: 36
**Salicaceae (1)**												
	Sp. 1	F	w	Manirosi		Tree	Contraception	Root	Decoction	Oral	**2**	M: 27
	(R54)			Pamaki			Wound healing	Fruit	Fresh	External		M: 29
**Sapindaceae (2)**												
	Sp. 1 (EL11)	F	w	Shiritsa		Shrub	To bathe babies	Leaves	Decoction	External	**1**	F: 45
	Sp. 2 (WA03)	F	w			Shrub	Gonorrhoea	Sap	Infusion	External	**1**	M: 29
**Sapotaceae (1)**												
	Micropholis venulosa (Mart. & Eichler) Pierre (LI12)	F	w	Shintsiquirishi		Tree	To strenghten the body	Bark, leaves and root	Decoction	Oral	**1**	F: 36
**Scrophulariaceae (1)**												
	Scoparia dulcis L. (0912)	H	w	Pichana	Machangará	Herb	To prevent hair loss	Leaves and stem	Decoction	External	**1**	F: 24

**Smilacaceae (1)**											
Smilax kunthii Killip & C.V. Morton (R44)	F	w	Zarzaparrilla		Liana	Browses and swellings	Tuberous rhizome	Fresh	External		M: 27
**Solanaceae (16)**											
Brunfelsia grandiflora D. Don	H	c	Sira	Chirisanango	Shrub	Stomach ache	Root	Cold water extract	Oral	**7**	F: 36
(1001, 2905, 4008)						Sorcery	Root	Steam bath	External		M: 33<br>F: 36
							Leaves	Decoction	External		M: 50
							Leaves and root	Decoction	Oral		M: 38
						Cold	Leaves	Alcohol extract	Oral		M: 69
						Osteoarthritis	Leaves	Alcohol extract	Oral		M: 69
								Decoction	External		F: 24
						Mal aire	Leaves	Decoction	Oral		M: 50
									External		M: 50, 38
Cestrum hediondinum Dun. (0607, 2904, R91)	H, F	c, w	Amposhiniaki	Hierba santa	Small tree	Fever	Leaves	Infusion	External	**17**	M: 33<br>F: 46, 24, 42, 30
								Decoction	Oral		F: 36<br>M: 57
			Shitsiroshi						External		F: 36, 71<br>M: 57, 32
								Cold water extract	Oral		F: 24, 27, 55, 46
									External		M: 27, 38<br>F: 35
						Stomach ache	Leaves	Decoction	Oral		M: 26
						Headache	Leaves	Decoction	External		M: 50<br>F: 71
								Cold water extract	External		M: 55
						Mal aire	Leaves	Decoction	External		M: 50<br>F: 45
						Sorcery	Leaves	Decoction	External		M: 50
						Malaria	Leaves	Decoction	Oral		F: 71

Nicotiana tabacum L. (0601)	H	c	Sheri	Tabaco	Herb	Insect bites	Leaves	Heated	External	**10**	F: 46, 25<br>M: 26, 27
						Used by tobacco healer	Leaves	Syrup	Oral		F: 30<br>M: 26, 27, 38, 50, 69
						Mal aire	Leaves	Decoction	External		M: 50<br>F: 25
								Steam bath	External		M: 50<br>F: 25, 28
						Headache	Sap	Heated	Eye		F: 42
Nicotiana sp. (PE28)	F	w		Tabaco del monte	Herb	Browses and swellings	Leaves	Emplast	External	**1**	M: 55
Physalis pubescens L. (WA5)	F	w	Kapuli		Herb	Stomach ache	Leaves	Decoction	Oral	**1**	M: 29
						Diarrhoea	Leaves	Decoction	Oral		M: 29
						Stomach parasites	Leaves	Decoction	Oral		M: 29
Solanum americanum Mill. (PL2)	B	w	Tzenirishi		Herb	Wound healing	Leaves	Heated	External	**6**	F: 36, 35, 24
								Fresh	External		M: 50
						Headache	Leaves	Fresh	Eye		M: 69
						Diarrhoea	Leaves	Cold water extract	Oral		M: 38
Solanum mammosum L. (3004)	H	c	Shinkirontsa		Shrub	Headache	Leaf sap	Fresh	Eye	**1**	M: 50
Solanum sp.1 (CA1)	F	w	Mapochashi		Herb	Arcoiris	Leaves	Fresh	External	**1**	F: 35
Solanum sp.2 (FL8)	F	w		Camotilla	Herb	Headache	Leaves	Fresh	Eye	**1**	F: 71
Solanum sp. 3 (R5)	F	w	Tsemenkirish		Small tree	Headache	Root	Fresh	Eye	**4**	M: 27, 55<br>F: 71
						Burned skin	Root	Fresh	External		M: 29

Sp. 1	H	c		Hierba santa	Small tree	Headache	Leaves	Decoction	Oral	**1**	F: 25
(3702)									External		F: 25
Sp. 2	F	w	Kintiaishi		Herb	To bathe babies	Leaves	Decoction	External	**2**	F: 45
(AQ15)			Pijojoshi			Wound healing	Stem	Cold water extract	External		M: 50
						To bathe babies	Leaves	Decoction	External		M: 50
Sp. 3	F	w	Pijoro		Shrub	Wound healing	Leaves	Fresh	External	**1**	M: 19
(ROM8)											
**Tiliaceae (3)**											
Heliocarpus americanus L.	F	w	Shinti	Huampo blanco	Tree	Gastritis	Bark	Decoction	Oral	**8**	M: 27, 29
(R14)			Shintse			Internal inflammations	Bark	Decoction	Oral		M: 27
						Stomach ache	Bark	Decoction	Oral		F: 55
						Prostate	Bark	Decoction	Oral		M: 50
						To give birth rapidly	Bark	Decoction	Oral		M: 29
						Kidney-complaints	Bark	Decoction	Oral		M: 32<br>F: 71, 55
						Ovary inflammation	Bark	Decoction	Oral		M: 55
						Inflammation of urinary duct	Bark	Decoction	Oral		F: 36
Triumfetta calycina Turcz.	H	w		Amor seco	Shrub	Inflammation of urinary duct	Root	Decoction	Oral	**1**	F: 25
(0913)						Kidney-complaints					F: 25
Triumfetta lappula L.	F	w	Tsireroki	Pega pega	Shrub	To enhance women fertility	Root	Decoction	Oral	**9**	M: 27, 32
(R11)						Ovary inflammation	Stem and root	Cold water extract	Oral		M: 55
						Haemorrhage	Leaves and root	Fresh	Oral		M: 50
							Bark	Decoction	Oral		F: 46
						Kidney-complaints	Bark	Decoction	Oral		F: 36, 71
							Root	Fresh	Oral		F: 55
						Stomach ache	Root	Decoction	Oral		F: 45
**Urticaceae (6)**											

Urera cf. baccifera	F	w	Tsiretishi	Chalanca macho	Herb	Mal aire	Leaves	Steam bath	External	**11**	M: 27<br>F: 71
(R90, R92)							Leaves and stem	Fresh	Oral		F: 46
								Decoction	External		F: 45
			Ishtashipana	Chalanca blanca		Kidney-complaints	Leaves and root	Decoction	Oral		F: 36
			Ishtashiretsi			Seeing shadows	Stem	Fresh	Eye		F: 55
			Hirina	Chalanca morada		Headache	Root	Fresh	Eye		F: 71
				Chalanca hembra		Pain in the muscles after work	Whole plant	Steam bath	External		M: 50
						General not well being	Root	Decoction	Oral		M: 69
								Decoction	External		M: 50
						Cramps	Leaves	Fresh	External		M: 38<br>F: 30
Urera cf. capitata	F	w	Poronketo	Chalanca simple	Herb	Mal aire	Leaves	Steam bath	External	**7**	M: 27
(R95)						Heart-complaints	Stem	Fresh	Oral		F: 36
						Ovary inflammation	Leaves	Fresh	External		F: 71, 23
						General not well being	Leaves	Decoction	External		M: 50
						Cramps	Leaves	Fresh	External		M: 27
						Liver-complaints	Root	Decoction	Oral		F: 25
						Kidney-complaints	Root	Decoction	Oral		F: 25
Urera cf. caracasana<br>(PA02)	F	w	Kairinpiroki		Herb	Uta (Leishmaniasis)	Sap	Fresh	External	**1**	M: 38
Urera cf. eggerii<br>(CA07)	F	w			Herb	Violent men	Root	Fresh	Oral	**1**	F: 35
Urera laciniata Wedd. (R32, 2817)	F	w	Hirina	Chalanca de mula	Herb	Sorcery	Leaves and stem	Steam bath	External	**10**	M: 27
			Poronketo			Kidney-complaints	Root	Decoction	Oral		F: 36, 46
						Prostate	Root	Decoction	Oral		M: 55
						Cramps	Leaves	Decoction	External		F: 55<br>M: 50
						Sorcery	Leaves and root	Steam bath	External		F: 71, 32
						Mal aire	Leaves and stem	Decoction	External		F: 35

						Osteoarthritis	Root	Decoction	Oral		M: 29
						Fever	Leaves and root	Steam bath	External		F: 46
						Measles	Leaves	Decoction	External		F: 32
						To improve male libido	Root	Decoction	Oral		F: 32
Sp. 1 (R37)	F	w	Shiritsipana	Chalanca de mula	Herb	Seeing shadows	Stem	Fresh	Eye	**4**	M: 27
				Chalanca de monte		Mal aire	Leaves	Steam bath	External		F: 36
				Chalanca morada				Decoction	External		F: 36<br>M: 29, 57
						Cramps	Leaves	Decoction	External		M: 57
**Verbenaceae (10)**											
Lantana camara L. (0901)	H	c	Pijoro		Herb	Fever	Leaves	Decoction	External	**1**	F: 24
						Headache	Stem	Fresh sap	Eye		F: 24
Lantana sp. (2808)	H	c	Ojecharishe		Herb	Arcoiris	Leaves	Decoction	External	**1**	F: 30
Lippia alba (Mill.) M.E.Br. (0115)	H	c		Oregano de selva	Herb	Mal aire	Leaves	Decoction	External		F: 71
						To give birth rapidly	Leaves	Infusion	Oral		F: 71
Lippia sp. (0116)	H	c			Vine	To improve male libido	Leaves	Decoction	External	**1**	F: 71
Phyla sp. (PL05)	B	w		Verbena	Herb	Malaria	Leaves	Decoction	Oral	**3**	F: 35, 36, 25
Priva lappulacea (L.) Pers. (4009, 4002)	H	w	Tsireroqui		Herb	Kidney-complaints	Leaves	Decoction	Oral	**1**	F: 32
Stachytarpheta cayennensis (Rich.) Vahl (2805)	H	w			Herb	Snake bites	Leaves	Decoction	External	**1**	M: 69
Stachytarpheta sp. (2918)	H	w	Tsireroqui	Pega pega	Herb	To be faithful to the partner	Leaves	Fresh	External	**1**	M: 33

Verbena sp. (2920, 3701)		H	c	Kepishiri	Verbena	Herb	Malaria	Leaves	Decoction	Oral	**13**	M: 33, 38
								Leaves and stem	Fresh	Oral		M: 27, 26<br>F: 25
							Stomach ache	Leaves	Decoction	Oral		M: 50, 69, 27, 27<br>F: 30, 28
								Root	Decoction	Oral		F: 38
							Diarrhoea	Leaves	Fresh	Oral		F: 42, 24
							To bathe babies	Leaves	Decoction	External		F: 42
Sp. 1	(WI18)	F	w	Ishiwiguionkishi samani		Herb	Contraception	Flower	Decoction	Oral	**1**	M: 32
**Vitaceae (2)**												
Cissus gongylodes (Baker) Burch. (0106, R42)		H	c		Suelda suelda	Vine	Browses and swellings	Stem	Emplast	External	**2**	F: 71
									Decoction	Oral		F: 71
							Bone fractures		Emplast	External		M: 27
									Decoction	Oral		M: 27
							Joint dislocations		Emplast	External		M: 27
									Decoction	Oral		M: 27
Cissus sp. (2902)		H	c	Nodo-nodo		Vine	Bone fractures	Stem	Emplast	External	**1**	F: 33
**Zingiberaceae (4)**												
Costus sp.	(FL23)	F	w	Kauo		Herb	Mal aire	Leaves and stem	Decoction	External	**4**	F: 71<br>M: 69, 50
										Oral		M: 50
Sp. 1 (0703)		H	c			Herb	Arcoiris	Stem	Fresh	External	**1**	F: 21
Sp. 2 (AQ1)		F	w	Akorishi		Herb	To bathe babies	Leaves and stem	Decoction	External	**1**	F: 45

Sp. 3	(R24)	F	w	Porenki		Herb	To strenghten newborn babies	Leaves	Decoction	External		M: 27

Not identified:

Sp. 1 (LI17)		F	w	Kachonpitoki		Herb	Stomach acidity	Stem	Decoction	Oral	**1**	F: 36
Sp. 2 (0112)		H	w	Pinitsi		Herb	Headache	Stem	Fresh	Eye	**1**	F: 71
Sp. 3 (0117)		H	w	Pinitsi		Herb	Mal aire	Leaves	Steam bath	External	**1**	F: 71
Sp. 4 (0903)		H	w	Nihashi		Herb	Chacho	Leaves	Decoction	Oral	**1**	F: 25
Sp. 5 (2804)		H	w	Achorite		Herb	Cramps	Leaves	Decoction	External	**1**	M: 38
Sp. 6 (2807)		H	w	Tevarishe		Herb	Skin rashes due to allergy	Leaves	Decoction	External	**1**	M: 38
Sp. 7 (2816)		F	w	Shiretsiwenki		Herb	Mal aire	Leaves	Decoction	External	**1**	F: 32
Sp. 8 (2910)		H	w	Pinitsi	Pusanga	Herb	To give birth without pain	Leaves	Decoction	Oral	**1**	F: 33
Sp. 9 (2922)		H	w	Poroquish		Shrub	Wound healing	Leaves	Emplast	External	**1**	F: 33
Sp. 10 (2929)		H	w	Poinirotsa		Herb	Stomach ache	Root	Decoction	Oral	**1**	F: 33
Sp. 11 (3001)		H	c	Tantashi		Herb	To prevent hair loss	Leaves	Decoction	External	**1**	M: 50

Sp. 12 (3008)		H	w	Tampiashi	Herb	To get ones spirit back	Leaves	Ashes	External	**1**	M: 50
Sp. 13 (AQ13)		F	w		Herb	Swollen feet	Leaves	Boiled emplast	External	**1**	F: 46
Sp. 14 (AQ4)		F	w	Kosinthitishi	Herb	Headache	Root	Decoction	Oral	**1**	F: 46
Sp. 15 (CA3)		F	w		Herb	Headache	Leaves	Fresh	Eye	**1**	F: 35
Sp. 16 (DH3)		F	w	Tonkeroshe	Herb	Mal aire	Leaves	Decoction	Oral	**1**	M: 57
Sp. 17 (DH06)		F	w		Herb	To improve male libido	Latex	Fresh	Oral	**1**	M: 57
Sp. 18 (EL2)		F	w		Herb	Snake bites	Leaves	Boiled emplast	External	**1**	F: 45
Sp. 19 (FL1)		F	w		Herb	Mal aire	Leaves and stem	Steam bath	External	**1**	F: 71
Sp. 20 (FL4)		F	w		Herb	Uta (Leishmaniasis)	Latex	Fresh	External	**1**	F: 71
Sp. 21 (FL15)		F	w	Pariuana	Herb	Haemorrhage	Leaves	Steam bath	External	**1**	F: 71
Sp. 22	(FL26)	F	w	Cortadillo	Herb	To abort	Leaves	Cold water extract	Oral	**1**	F: 71
Sp. 23 (FL6)		F	w		Herb	Prostate	Root	Decoction	Oral	**1**	F: 71

Sp. 24 (FL9)	F	w			Herb	Sorcery	Leaves	Steam bath	External	**1** F: 71
Sp. 25 (IS1)	F	w	Poi		Herb	Browses and swellings	Tuber	Emplast	External	**1** F: 55
Sp. 26 (LI18)	F	w	Tsemenquirish		Shrub	Headache	Root	Fresh	Eye	**1** F: 36
							Leaves	Fresh	Eye	F: 36
Sp. 27 (LI19)	F	w			Herb	Wound healing	Latex	Fresh	External	**1** F: 36
Sp. 28 (LI2)	F	w	Tsirinishi		Herb	To extract caried teeth	Stem	Fresh	External	**1** F: 36
Sp. 29 (LI21)	F	w	Shipetashi		Herb	To strenghten the body	Leaves	Decoction	External	**1** F: 36
Sp. 30 (LI24)	F	w	Tsampobatashi		Herb	Arcoiris	Leaves	Fresh	External	**1** F: 36
Sp. 31 (LI7)	F	w	Tsireroquishi	Chanca piedra	Herb	Menstruation pain	Leaves	Decoction	Oral	**2** F: 36
						Kidney-complaints	Leaves	Decoction	Oral	F: 36
				Pega pega		Stomach ache	Leaves, stem and root	Decoction	Oral	M: 50
Sp. 32 (LI8)	F	w			Herb	Haemorrhage	Leaves	Decoction	Oral	**1** F: 36
Sp. 33 (PE11)	F	w	Kitoriki		Tree	Chickenpox	Leaves	Decoction	External	**1** M: 55
							Bark	Decoction	External	M: 55
Sp. 34 (PE13)	F	w	Puchonaki		Tree	Asthma	Leaves	Decoction	Oral	**1** M: 55
Sp. 35 (PE14)	F	w	Tavaironkareki		Shrub	To bathe babies	Leaves	Decoction	External	**1** M: 55
						To make babies walk fast	Leaves	Decoction	External	M: 55

						To strenghten newborn babies	Leaves	Decoction	External		M: 55
Sp. 36 (PE21)	F	w	Ungurabe		Tree	Prostate	Seed	Decoction	Oral	**1**	M: 55
Sp. 37 (PE27)	F	w	Camarishi		Herb	Sorcery	Leaves	Decoction	External	**1**	M: 55
Sp. 38	F	w		Papaya del monte	Herb	Fever	Leaves	Decoction	External	**4**	M: 55
(PE3)						Emesis	Leaves	Decoction	External		M: 55
				Matico del monte		Mal aire	Leaves	Decoction	External		M: 69, 50
			Mapochashi			Pokio	Leaves	Heated emplast	External		F: 38
Sp. 39	F	w	Shivitha		Liana	To improve male libido	Bark	Decoction	Oral	**5**	M: 27<br>F: 71
(R67)							Latex	Fresh	Oral		F: 36<br>M: 29
						Hernia	Latex	Fresh	External		M: 19
Sp. 40	F	w	Katsa		Liana	Kidney-complaints	Bark	Decoction	Oral	**8**	M: 27, 55
(R80)			Chokirotsa			Influenza	Bark	Decoction	Oral		M: 29
						Cough	Bark	Decoction	Oral		M: 29
				Nudo nudo		Osteoarthritis	Stem	Decoction	Oral		F: 46
						Hernia	Leaves	Decoction	Oral		M: 38
							Stem	Decoction	Oral		M: 38
						Scapular arthritis	Stem	Boiled emplast	External		M: 57
						To enhance hair growth	Bark	Decoction	External		F: 55
			Ovavirotsa			Joint dislocations	Stem	Emplast	External		F: 71
Sp. 41	F	w	Cuatro esquinas		Herb	Malaria	Leaves	Decoction	Oral	**6**	M: 27
(R94)			Shawetashi			Cholera	Leaves	Decoction	Oral		M: 27
						Liver-complaints	Leaves	Decoction	Oral		F: 36
						To abort	Leaves	Decoction	Oral		F: 36
						Stomach parasites	Leaves	Fresh	Oral		M: 55<br>F: 45
						To provoke emesis	Leaves ans stem	Decoction	Oral		F: 55

							Hepatitis	Leaves	Fresh	Oral		F: 71
Sp. 42	(WI8)	F	w	Matsikishi		Herb	To strenghten newborn babies	Leaves	Decoction	External		M: 32
Sp. 43	(PE17)	F	w	Shintsiquerishi		Herb	To strenghten the body	Leaves	Decoction	External	**1**	M: 55
								Root	Decoction	Oral		M: 55
Sp. 44	(PL09)	B	w	Shancorotsinco		Herb	Liver-complaints	Leaves	Infusion	Oral	**7**	F: 25, 35, 36
										External		F: 25, 35, 36
							To prevent ageing	Leaves	Infusion	Oral		F: 25, 35, 36
							Joint dislocations	Leaves	Infusion	External		M: 26
				Chankoroti	Verdulaga		To give birth rapidly and not feel the pain	Leaves and root	Decoction	Oral		M: 27
				Lechugia			Liver-complaints	Leaves	Decoction	Oral		M: 50
				Shankorotinko			To gain weight	Root	Decoction	Oral		F: 23
Sp. 45		F	w	Kipishiri		Herb	Stomach parasites	Root	Cold water extract	Oral	**1**	F: 36
(LI13)												

DIVISION PTERIDOPHYTA

Adiantaceae (1)

Adiantum cayennense Willd. ex Klotzsch (3005)	H	w	Takére ki maranki		Herb	Snake bites	Leaves	Emplast	External	**1**	M: 50

Aspleniaceae (2)

Asplenium serratum L.	F	w	Yashe attara tarazipini	Lengua de perro	Herb	Internal pain 'vaso'	Leaves	Decoction	Oral	**4**	M: 27
(R83)			Mainishi			Stomach ache	Leaves	Decoction	Oral		F: 71
									External		F: 71
						To prevent ageing	Leaves	Fresh	Oral		F: 35
						Skin spots	Leaves	Fresh	External		F: 36
Asplenium sp.	F	w	Manihi		Herb	Insect bites	Leaves	Fresh	External	**3**	M: 27

(R70)						Stomach ache	Leaves	Decoction	Oral		F: 36
								Steam bath	External		F: 36
			Manitishi			Mal aire	Leaves	Steam bath	External		F: 55
**Blechnaceae (2)**											
Salpichlaena volubilis (Kaulf.) J. Sm.	F	w	Mainishi		Herb	Sleep disorders	Leaves	Decoction	External	**5**	M: 55
(PE32, IS7)						To prevent ageing	Leaves	Fresh (Chewed)	Oral		F: 55
Blechnum occidentale L.	F	w	Tsombishi		Herb	Fatigue suppressor	Leaves	Fresh (Chewed)	Oral		F: 46
(AQ7)						Skin spots	Leaves	Fresh	External		F: 42
			Tsirompishi	Chaca chaca		To attract men	Leaves	Decoction	External		M: 27
				Pusanga							
**Cyatheaceae (1)**											
Cyathea multiflora Sm.	F	w	Irakumaníti	Puño de tigre	Arborescent fern	Wound healing	Top of caudex	Fresh	External	**9**	M: 27
(R15)			Tiniro			Hernia	Top of caudex	Fresh	External		M: 50
						Ovary inflammation	Top of caudex	Decoction	Oral		M: 29
						To make babies walk fast	Top of caudex	Decoction	External		M: 32, 38
						To lift up testicles	Top of caudex	Decoction	External		M: 55
								Fresh	External		F: 71
						Kidney-complaints	Top of caudex	Decoction	Oral		F: 36
						Cancer	Top of caudex	Decoction	Oral		F: 36
						Browses and swellings	Top of caudex	Fresh	External		F: 55
						Liver-complaints	Top of caudex	Decoction	Oral		F: 71
**Davaliaceae (1)**											
Nephrolepsis sp.	F	w			Herb	Acne	Leaves	Fresh	External	**2**	M: 19
(ROM5)				Pusanga		To attract the other sex	Leaves	Alcohol extract	External		M: 69
**Dryopteridaceae (2)**											
Cyclopeltis semicordata (Sw.) J. Sm.	F	w	Marankishi		Herb	Snake bites	Leaves	Emplast	External	**1**	M: 55
(PE6)											

Sp. 1 (WI10)	F	w	Lekintsishi		Herb	Emesis	Sap	Fresh	Oral	**1**	M: 32
**Equisetaceae (1)**											
Equisetum giganteum L. (PL6)	B	w		Cola de caballo	Herb	Kidney-complaints	Leaves and stem	Infusion	Oral	**8**	M: 69<br>F: 36, 35, 24,38
						Liver-complaints	Leaves and stem	Infusion	Oral		M: 69<br>F: 36, 35, 24
						Pains in the body	Leaves	Decoction	Oral		M: 50
			Sunkabina			Inflammation	Leaves	Decoction	Oral		M: 38
						Ulcers	Leaves	Decoction	Oral		M: 38
						Colics	Leaves	Decoction	Oral		M: 38
						Pokio	Leaves and stem	Decoction	Oral		F: 28
						Arcoiris	Leaves and stem	Decoction	Oral		F: 28
						Chacho	Leaves and stem	Infusion	Oral		F: 24
			Ishipatonashi			Prostate	Leaves and stem	Infusion	Oral		F: 24
**Gleicheniaceae (1)**											
Dicranopteris pectinata (Willd.) Underw. (PE29)	F	w	Chamantoza		Herb	To strenghten newborn babies	Leaves	Decoction	External	**1**	M: 55
**Pteridaceae (1)**											
Pteris sp. (PE18)	F	w	Tsiowinkishi		Herb	Epilepsy	Leaves	Decoction	External	**1**	M: 55
**Selaginellaceae (1)**											
Selaginella conduplicata Spring (R84)	F	w	Tsirishi		Herb	To relax the body	Leaves and stem	Decoction	External	**5**	M: 27
						To bathe babies	Leaves and stem	Decoction	External		M: 55<br>F: 55, 71
						To prevent hair loss	Leaves and stem	Decoction	External		M: 38
**Not identified:**											
Sp. 1	F	w			Herb	Snake bites	Leaves	Decoction	External	**1**	F: 46

(AQ8)											
Sp. 2	(PE23)	F	w	Kushipirinkeshi	Herb	Snake bites	Leaves	Emplast	External	**1**	M: 55

[a] Habitat types: home gardens, H; forest, F; river banks, B. [b] Status: (w) wild; (c) cultivated.

[c] No. of taxa. [d] No. of voucher specimens.

Exotic species.
