# Supplementary material for: Asháninka medicinal plants: a case study from the native community of Bajo Quimiriki, Junín, Peru
Source: J Ethnobiol Ethnomed. 2010 Aug 13;6:21. doi: 10.1186/1746-4269-6-21 (PMC2933607; doi:10.1186/1746-4269-6-21)
Supplement: Additional file 2 — List of Asháninka informants. The list provides the names of the inhabitants of the Asháninka Native Community Bajo Quimiriki who participated in the various activities, sharing their knowledge on medicinal plants. [file 1746-4269-6-21-S2.PDF]

Additional file 2. List of the inhabitants of the community of Bajo Quimiriki who participated in the various activities

Establishment of the forest track

Eduardo Hereña Quinchocre
German Benavides Shivanco
Luis Santico Hereña
Rolando Peralta Quinchocre

Walks on the river banks

Beatriz Peralta Quinchocre
Carolina Maximo Atilio
Lidia Martinez Paredes

Forest walks

Anna Leiba Quinchocre
Carolina Maximo Atilio
Daniel Orestes
Elena Quinchocre
Flora Hereña Manunca
Isabel Quinchocre Leiva
José Atilio Peralta Contreras
Lidia Martinez Paredes
Pedro Maximo Atilio
Romer Maximo Sebastian
Wilmer Romero Ignacio
Woker Martinez

Cross-check

Xenia Barona Santiago
Flora Samaniego Quinchisa
Yanet Hereña
Beatriz Peralta Quinchocre
Roxana Aurelio Paulina
Eima Sebastian Ramos
Enderson Poma
Jaime Cardenas
Henry Poma Paulina
Luis Santico Hereña
Manuel Hereña Campos
Rolando Peralta Quinchocre

Household interviews & collection of medicinal plants in the homegardens

Anna Leiba Quinchocre
Basilio Mejia Lopez
Beatriz Peralta Quinchocre
Bersilia Benavides
Carlos Camacho Camacho
Daniel Orestes
Ester Martinez Paredes
Flora Manonga Hereña
German Benavides Shivanco
Gilmer Armando Segura Puchoc
Henry Poma Paulina
Janet Quinchocre
Janeth Martinez Paredes
Kelly Andres Ramos
Lidia Martinez
Veronica Benavides
Vilma Martinez Paredes
Wilmer Romero Ignacio
Yolanda Suarez
